# *N*′-(Furan-2-ylmethylene)-2-hydroxybenzohydrazide and its metal complexes: synthesis, spectroscopic investigations, DFT calculations and cytotoxicity profiling

**DOI:** 10.1186/s13065-023-01098-8

**Published:** 2024-01-28

**Authors:** Nasser M. Hosny, Ghada Samir, Mohamed H. Abdel-Rhman

**Affiliations:** 1 Chemistry Department, Faculty of Science, Port Said University, P.O. Box 4252, Port-Said, Egypt; 2 Chemistry Department, Faculty of Science, Mansoura University, Mansoura, Egypt

**Keywords:** Schiff’s base, Metal complexes, DFT computation, Spectral studies, Cytotoxicity

## Abstract

The ligand, *N′*-(furan-2-ylmethylene)-2-hydroxybenzohydrazide (H_2_L), was synthesized characterized through various spectral studies which cleared out that the free ligand existed in keto form. The ligand upon reaction with Cu(II), Co(II), Ni(II) and Zn(II) acetates yielded complexes with stoichiometric ratio 1:2 (M:L) which has been validated through the elemental and mass spectral measurements. The IR and NMR spectral studies of the isolated complexes disclosed that the ligand chelated to metal ion in mononegative bidentate fashion via the azomethine nitrogen and deprotonated enolized carbonyl oxygen. Moreover, the DFT quantum chemical calculations designated that the ligand and Ni(II) complex exhibited the highest and lowest values of HOMO, LUMO energies and HOMO-LUMO energy gap, respectively. Furthermore, the in vitro cytotoxic activity towards HePG-2 and HCT-116 cell lines of the isolated compounds was investigated and the data cleared out that the ligand was more potent than the metal complexes.

**Supplementary Information:**

The online version contains supplementary material available at 10.1186/s13065-023-01098-8.

## Introduction

Since their first discovery by Hugo Schiff in 1864, the Schiff bases have been received significant attention owing to their facile synthesis by condensation reaction of primary amines with carbonyl compounds, such as aldehydes or ketones [1]. In coordination chemistry, Schiff base have been considered as a unique class of ligands due to their incorporate diverse donor groups and exhibiting good flexibility [2, 3]. Consequently, their metal complexes are significant because of its stability, capability to form metal chelates in mono-, bi- and polydentate manner, and wide range biological applications [4–9]. Additionally, several Schiff bases were exhibited potent antibacterial, antifungal, anticancer and diuretic activities and were widely utilized in food and dye industry, analytical chemistry, catalysis and agrochemical activities [10]. The biological activities of these compounds may be attributed to the presence of azomethine nitrogen, C=N, which serves as binding site for metal ions to interact with various biomolecules like proteins and amino acids [11].

Among the Schiff bases, heterocyclic derivatives possessed nitrogen and oxygen atoms as electron donors were heavily studied because of their therapeutic potentials against certain types of tumors [12, 13]. For example, the furan Schiff base derivatives and its metal complexes behaved as bidentate ligands via azomethine-N and furanyl-O which were the sites potentially responsible for the enhancement of biological activity [14]. Correspondingly, the furan-based Schiff base derivatives exhibit substantial antituberculosis, anti-inflammatory, antibacterial, antifungal and anticancer activities [15–18].

As well, the benzohydrazide derivatives and its metal complexes have been employed in many biological applications, as anti-inflammatory, antibacterial, and anticancer agents. The significant biological activity and extensive range of uses of benzohydrazide may be attributed to the presence of its potential keto-enol tautomeric forms, which arise from the electron delocalization between the adjacent amine and carbonyl groups [8, 19–22].

In contrast, the Cu(II), Co(II), Co(II), and Zn(II) complexes were examined for their adjustable electrical and spectroscopic characteristics, as well as their diverse structural functionality, which is essential for targeted applications. Moreover, they play a crucial role as an essential component in several enzymes, including urease and hydrogenases [23]. Furthermore, the metal-based medications were effectively capable to pass through the microbial membrane and exhibit strong binding affinities towards the genetic materials (RNA or DNA) of these pathogenic microorganisms [12, 13, 24].

Therefore, in continuation of our former endeavors to developing hydrazide-based hybrids and their transition metal complexes that may have anticancer activity [25–31], the present study presents the synthesis, characterization and anticancer application of the newly synthesized ligand, *N*′-(furan-2-ylmethylene)-2-hydroxybenzohydrazide, and its Cu(II), Co(II), Ni(II) and Zn(II) complexes.

## Experimental

### Materials and instruments

Furan-2-carbaldehyde (99%), 2-hydroxybenzohydrazide (99%) and metal acetate salts were of analytical grade (A. R. from Sigma-Aldrich or Merck). Fetal Bovine serum was purchased from GIBCO, UK, while the cell lines, hepatocellular (HePG-2) and colon (HCT-116) carcinoma were obtained from ATCC, Egypt. Both tetrazolium bromide (MTT) and RPMI-1640 medium were brought from Sigma Co., USA.

The carbon, hydrogen and nitrogen contents were determined on CHN analyzer Perkin-Elmer model 2400. The metals content were determined by standard methods [32]. The Thermo-Nicolet IS10 spectrometer employed for recording the FT-IR spectra, as KBr discs. The Unicam UV/Vis UV2 spectrometer used to measure the electronic spectra in 1 cm silica cells. The ^1^H NMR spectra were recorded on Bruker Ascend spectrometer 400 MHz. The Electron spin resonance spectra were recorded at room temperature on Brucker E 500 ESR spectrometer operating at 9.808 GHz, 100 kHz field modulation from 480 to 6480 Gauss in a 2 mm quartz capillary. TGA was measured on a Schimadzu model 50 instrument using heating rate 15 °C/min at 10 cm^3^/min nitrogen flow rate. The mass spectra were recorded on Varian MAT 311 spectrometer. Magnetic measurements were carried out on a Sherwood Scientific magnetic balance.

Mass spectra were made oninstrument.

### Synthesis of the ligand (H_2_L) and metal complexes

Furan-2-carbaldehyde (1.2 mL, 0.01 mol) was added drop wisely to ethanolic solution of 2-hydroxybenzohydrazide (1.52 g, 0.01 mol) then a few drops of glacial acetic acid were added. The reaction mixture was heated under reflux for 4 h where a faint brown precipitate of *N′*-(furan-2-ylmethylene)-2-hydroxybenzohydrazide was formed [33]. While hot, the precipitate was filtered off, washed successfully with ethanol, dried and recrystallized from hot ethanol (m.p.: 190 °C).

To ethanolic solution of H_2_L (0.02 mol), a water solution of the Cu(II), Co(II), Ni(II) or Zn(II) acetate (0.01 mmol) were added dropwise with stirring. The reaction mixture was refluxed for 2 h [22, 33]. The precipitates were filtered off, washed with hot ethanol followed by diethyl ether and dried in a vacuum desiccator over anhydrous CaCl_2_.

### DFT computations

The computational study of the isolated ligand (H_2_L) and its bivalent metal complexes was carried out using Gaussian 09 W [34] to explore their geometries. The geometrical optimization was proceeded for the neutral ground state in gas phase without any symmetry constraints [12, 13] at B3LYP level and 6–31++G(d,p) basis set [35–37]. The HOMO-LUMO illustrations were made by GaussView program [38].

### Cytotoxicity assay

The ligand and its metal complexes cytotoxic activity examination was performed by the MTT assay using doxorubicin as a standard anticancer drug for comparison. The cell lines were cultured in RPMI-1640 medium (10% fetal bovine serum). Antibiotics (100 units/mL penicillin and 100 µg/mL streptomycin) were added in a 5% CO_2_ at 37 °C incubator. The cells were incubated with the tested compounds for 24 h. After incubation time, 20 µL of tetrazolium bromide (MTT) solution (5 mg/mL) was incubated for four hours. Dimethyl sulfoxide (100 µL) was added to the formed purple formazan, then, the absorbance was measured at 570 nm using a plate reader (EXL 800, USA). The percentage of relative cell viability was calculated as (A_570_ of treated samples/A_570_ of untreated sample) × 100 [39, 40].

## Results and discussion

The ligand, *N′*-(furan-2-ylmethylene)-2-hydroxybenzohydrazide (H_2_L), elemental analyses (Table 1) revealed that it has C_12_H_10_N_2_O_3_ formula while its metal complexes had a 1:2 (M:L) stoichiometric, i.e., bis(*N*-(furan-2-ylmethylene)-2-hydroxybenzohydrazonate) cobalt(II), nickel(II), copper(II) and zinc(II) monohydrate. The metal complexes were soluble in DMF and DMSO only and exhibited molar conductivity, in DMSO, 3.2–8.1 Ω^−1^ cm^2^ mol^−1^ which indicated their non-electrolytic nature [41].

**Table 1:** Elemental analyses of H_2_L and the isolated metal complexes

Compound (formula; M.Wt. g/mol)	Color	M.P. (°C)	Elemental analysis % Found (calculated)			
			C	H	N	M
H_2_L (C_12_H_10_N_2_O_3_; 232.22)	Beige	190	62.86 (62.61)	4.48 (4.38)	12.61 (12.17)	–
[Co(HL)_2_] (C_24_H_18_CoN_4_O_6_;517.36)	Brown	> 300	55.41 (55.72)	3.83 (3.51)	11.06 (10.83)	11.55 (11.39)
[Ni(HL)_2_] (C_24_H_18_NiN_4_O_6_; 517.12)	Orange	> 300	55.48 (55.74)	3.72 (3.51)	11.18 (10.83)	10.90 (11.35)
[Cu(HL)_2_] (C_24_H_18_CuN_4_O_6_; 521.98)	Pale green	> 300	55.63 (55.23)	3.28 (3.48)	11.35 (10.73)	11.82 (12.17)
[Zn(HL)_2_](H_2_O) (C_24_H_20_ZnN_4_O_7_; 541.83)	Yellow	> 300	53.19 (53.20)	3.75 (3.72)	9.93 (10.34)	11.80 (12.07)

### FT-IR spectra

**Fig. 1:**
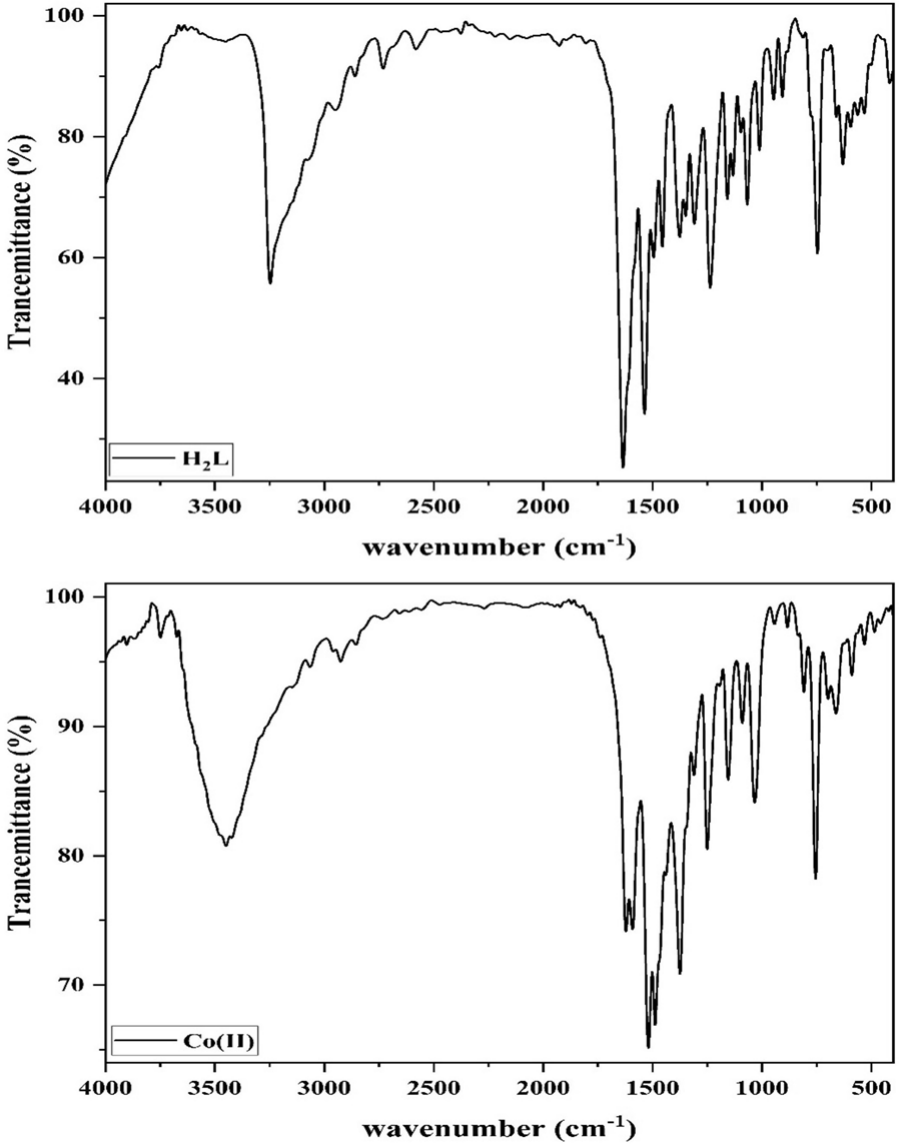
IR spectra of H_2_L and Co(II) complex

The ligand (H_2_L) has two possible tautomeric forms, keto and enol, as shown in Structure 1, therefore, its IR spectrum was carefully studied in order to determine in which form the ligand is existed. The spectrum displayed sharp band at 3248 cm^−1^ with a shoulder at 3142 cm^−1^ ascribed to the hydroxyl group ν(OH) [33, 42, 43] and hydrazonyl ν(NH) [33, 43], respectively. In addition, the strong band at 1635 cm^−1^ with shoulder at 1610 cm^−1^ were designated to the carbonyl ν(C=O) [33, 43] and azomethine ν(C=N) [33, 43], respectively. Furthermore, the aromatic ν(C=C) along with the furane ν_as_(C–O–C) and ν_s_(C–O–C) vibrations [44, 45] were observed at 1588, 1237 and 1017 cm^−1^, respectively (Fig. 1). Hence, the abovementioned findings implied that the carbonyl and hydrazonyl NH were present which advocated the keto-form of the free ligand. Also, the sharpness of the vibrational band ν(OH) along with the lower shift of ν(C=O) suggested their involvement in H-bond formation [42] (Table 2).

**Structure 1:**
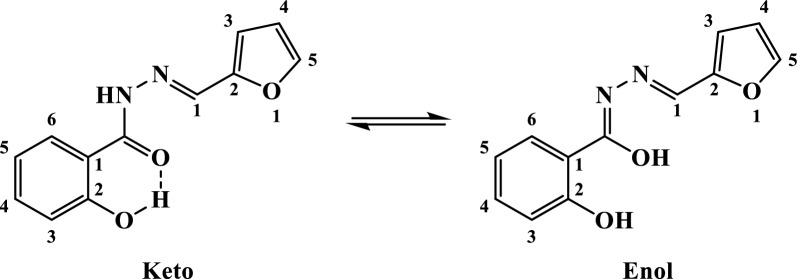
The possible tautomeric forms of the ligand

**Table 2:** Some IR bands of H_2_L and the isolated metal complexes

Bands	H_2_L	Cu(II)	Co(II)	Ni(II)	Zn(II)
ν(OH)_solv_	3248*	3449	3452	3448	3436
ν(NH)	3142	–	–	–	–
ν(CH)_Ar_	3072	3066	3062	3084	3063
ν(C=O)	1635	–	–	–	–
ν(C=N)	1610*	1620	1622	1628	1625
ν(C=C)	1588	1599	1592	1592	1589
Amide II	1537	1517	1519	1521	1520
ν_as_(C–O–C)_furan_	1237	1241	1257	1243	1242
ν(N–N)	1158	1147	1149	1151	1150
ν(C–O)	1066	1092	1090	1091	1085
ν_s_(C–O–C)_furan_	1017	1021	1035	1020	1019
ω(OH)	746	756	755	755	753
ν(M–O)	–	592	589, 532	565	588
ν(M–N)	–	475	484	488	445

*OH of the ligand

**Scheme 1:**
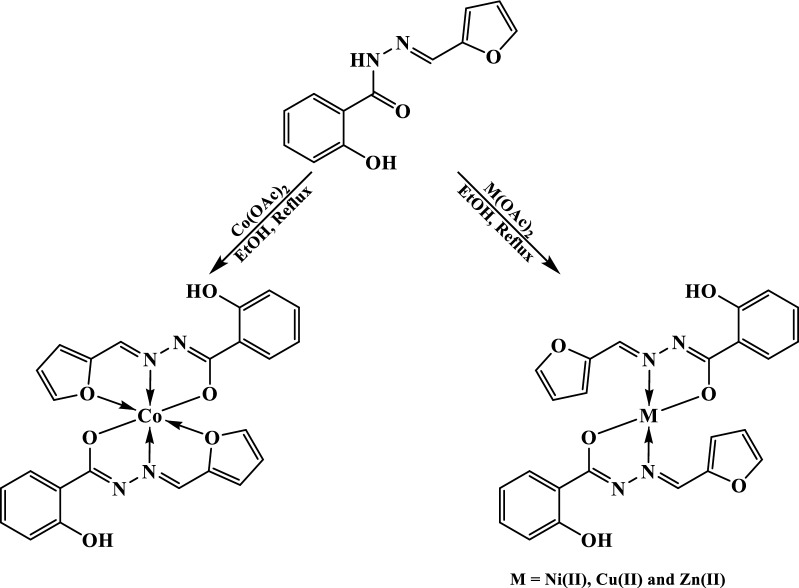
The suggested metal complexes structures

The spectra of metal complexes showed one broad band at 3436–3452 cm^−1^ which was attributed to the hydroxyl ν(OH) vibration where the position shift and broadness, with respect to that of the ligand, suggested that the hydroxyl group was free (no H-bond). Moreover, the two bands at 1620–1628 and 1589–1599 cm^−1^ regions were assigned to the azomethine ν(C=N) [33, 43] and aromatic ν(C=C) [33, 43], respectively (Fig. 1). Furthermore, the furane ν_as_(C–O–C) and ν_s_(C–O–C) vibrations [44, 45] were observed at about 1242 and 1020 cm^−1^, respectively, in all complexes except in Co(II) complex, where both were displayed at higher wavenumbers, 1257 and 1035 cm^−1^. Thus, the disappearance of both the carbonyl ν(C=O) and hydrazonyl ν(NH) with appearance of the ν(C=N) vibration band disclosed that the ligand existed in enol form. But, the absence of new band due to the newly formed OH group supported the deprotonation of such group on reaction with metal salt. The Amide II and ν(C=N) bands shift to lower position indorsed the ligand enolization and involvement of the azomethine groups in coordination to the metal ion. The higher shift of the furan bands in Co(II) complex was taken as evidence for participation of the furan oxygen in chelating the metal ion. Furthermore, the new bands at 565–592 and 445–488 cm^−1^ regions were attributed to ν(M–O) and ν(M–N) [46–48], respectively (Additional file 1: Fig. S1). The above-mentioned foundations suggested that the ligand chelated to the metal ion in enol form as mononegative bidentate or tridentate via C=N, the deprotonated enolic hydroxyl group or furan oxygen (Scheme 1).

### NMR spectra

**Fig. 2:**
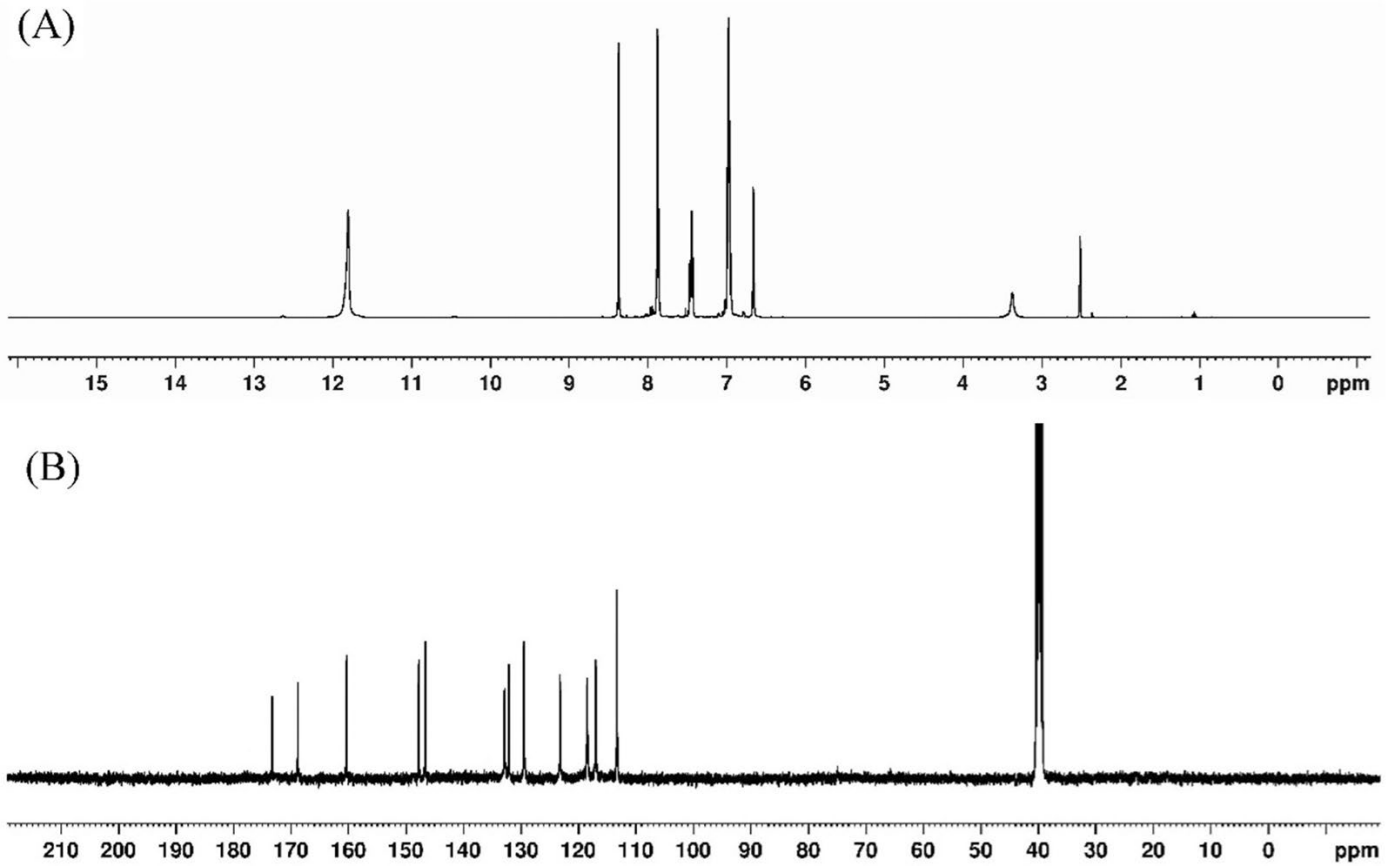
**A** ^1^H NMR of H_2_L and **B** ^13^C NMR spectrum of Zn(II) complex

The ligand ^1^H-NMR spectrum, in DMSO-d_6_, showed two multiplet signals at 6.65 and 6.97 ppm assigned to the protons of the furan at 3,4-positions and phenyl at 3,5-positions [42, 49], respectively. The triplet signal at 7.44 ppm was corresponded to phenyl at 4-position whereas the doublet signal present at 7.86 was attributed to the overlapping of phenyl-6 and furan-5 [42, 49]. Moreover, the two singlet signal at 8.37 and 11.80 ppm were ascribed to azomethine proton (HC=N) and both of phenolic OH and hydrazonyl NH [42, 49], respectively. The appearance of the phenolic OH overlapped with the NH proton confirms that the ligand exists in the keto form and involvement of OH in H-bond [29, 30] (Fig. 2A). On the other hand, the spectrum of Zn(II) complex, in DMSO*-*d_6_, showed a singlet signal at 13.98 ppm that was designated to the free phenolic proton (OH) [29, 30]. The other singlet signal at 8.59 ppm was ascribed to the azomethine proton (HC=N) [42, 50]. The appearance of only one signal due to the phenolic OH proton confirmed that it is free, no H-bond or metal binding, and the downfield shift of azomethine proton supported the participation of these groups in bonding to metal ion [42, 50]. The spectrum displayed the other bands at more or less the positions (Table 3).

**Table 3:** ^1^H and ^13^C-NMR data of H_2_L and Zn(II) complex

^1^H-NMR			^13^C-NMR		
Protons	H_2_L	Zn(II)	Carbons	H_2_L	Zn(II)
OH	11.80	13.98	C=O	165.0	169.0
NH	11.80	–	Ph-2	159.5	160.0
HC=N	8.37	8.59	Fur-2	149.7	147.9
Ph-6	7.86	7.74	Fur-5	145.9	146.7
Fur-5	7.86	7.52	C=N	138.3	173.4
Ph-4	7.44	7.34	Ph-4	134.3	132.9
Ph-3	6.97	6.89	Ph-6	128.9	132.0
Ph-5	6.97	6.89	Ph-5	119.4	123.2
Fur-3,4	6.65	6.66	Ph-3	117.7	118.6
			Fur-3	116.3	117.1
			Ph-1	114.6	129.6
			Fur-4	112.7	113.4

Additionally, the ^13^C-NMR spectrum of H_3_L showed two signals at 165.0 and159.5 ppm attributed to C=O andphenyl-2 [42, 49], respectively. The signals at 149.7, 145.9 and 138.3 ppm were assigned to the furan-2, furan-5 and azomethine HC=N [42, 49], respectively. The spectrum displayed several signals at 134.3, 128.9, 119.4, 117.7, 116.3, 114.6 and 112.7 ppm due to the phenyl-4, -6, -5, -3, furan-3, phenyl-1 and furan-4 [42, 49], respectively (Table 3). On contrary, the ^13^C NMR spectrum of Zn(II) complex showed two signals at 173.4, 169.0 and 160.0 ppm attributed to azomethine (HC=N), enolized carbonyl (N=C–O) and phenyl-2 carbons [42, 49], respectively.

### Mass spectra

**Fig. 3:**
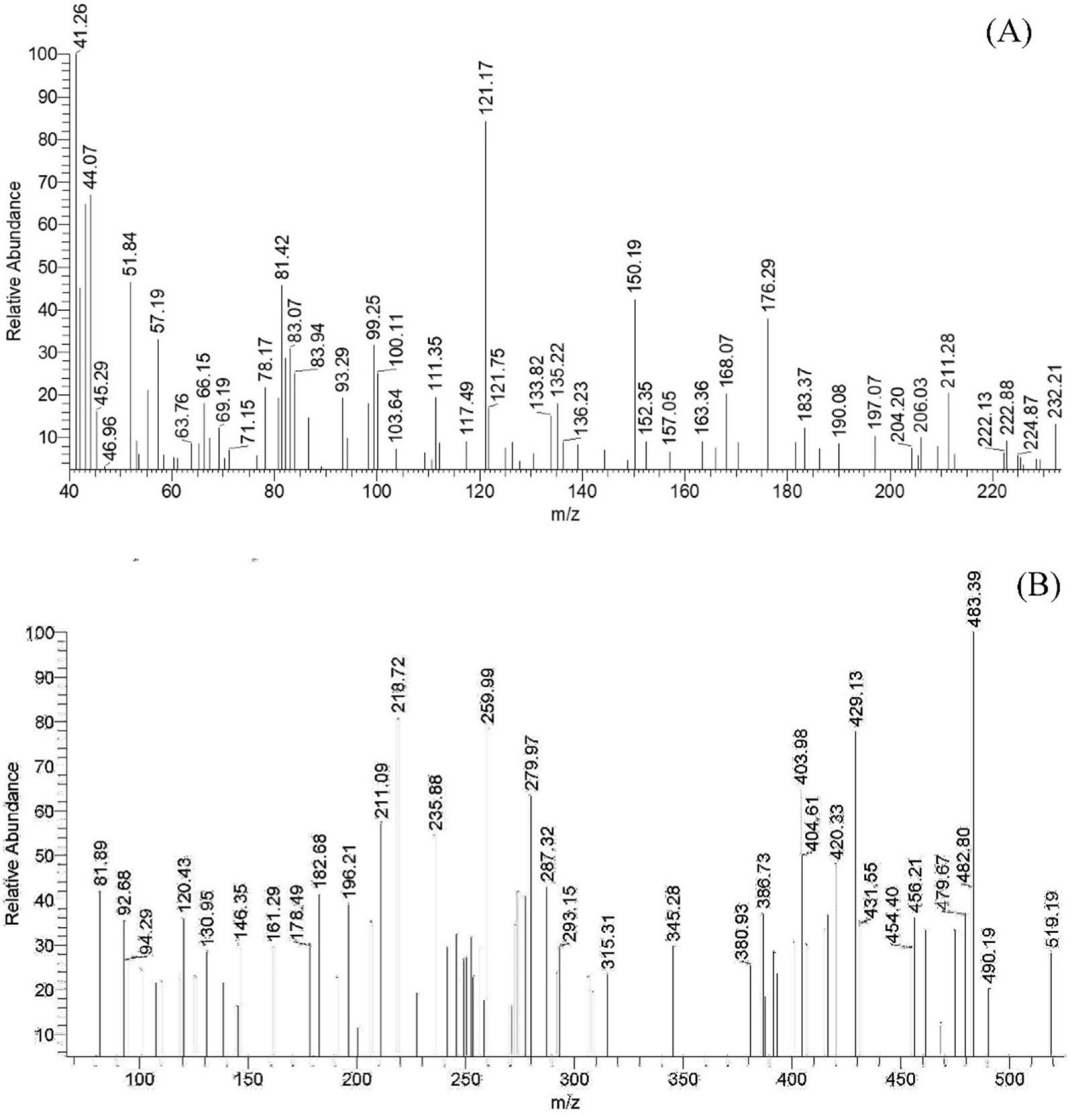
The mass spectrum of the ligand (**A**) and Co(II) complex (**B**)

**Scheme 2:**
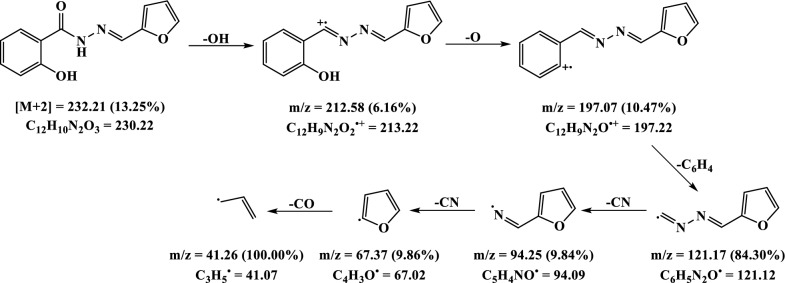
The suggested fragmentation pattern of the ligand

The mass spectrum of H_2_L exhibits the molecular ion peak at *m/z* = 232.2 corresponding to [M + 2] formula which in agreement with suggested ligand structure (C_12_H_10_N_2_O_3_; M.Wt. = 230.2) (Fig. 3A). Moreover, the spectrum displayed two peaks at *m/z* = 212.58 (6.16%) and 197.07 (10.47%) which resulted from loss of OH and O fragments, respectively. The peak observed at *m/z* = 121.17 (84.30%) was assigned to loss of phenyl ring to give C_6_H_5_N_2_O^**.**^ (F. Wt. = 121.12) which underwent two subsequent degradation steps by losing CN fragment in each, *m/z* = 67.37 (9.86%), leading to formation of furan radical (C_4_H_3_O^**.**^ = 67.02). Finally, the base beak at *m/z* = 41.26 (100.00%) was ascribed to the furan radical degradation by losing carbon monoxide moiety (CO) that led to formation of C_3_H_5_^**.**^ (F. Wt. = 41.07) (Scheme 2).

**Scheme 3:**
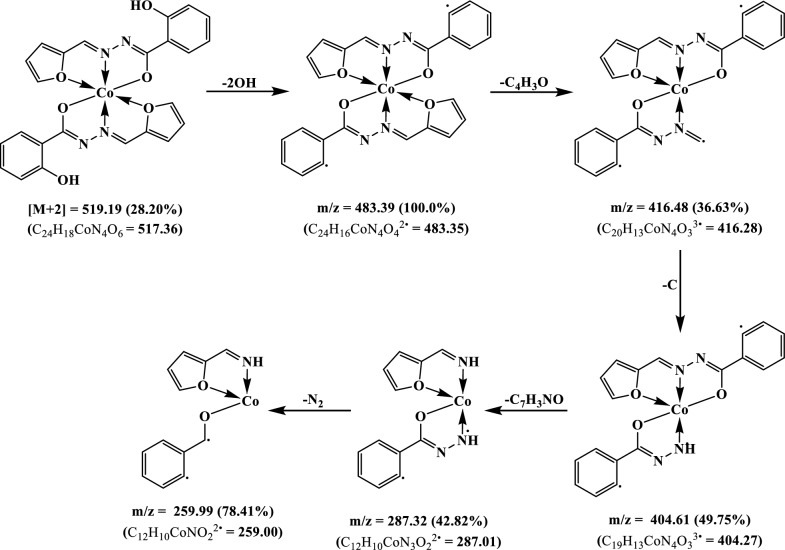
The suggested fragmentation pattern of Co(II) complex

On the other hand, the mass spectra of the metal complexes presented good evidence of the suggested structure as they displayed molecular ion peak at *m/z* = 521.33, 516.72 and 559.47 for Cu(II) (M. Wt. 521.98), Ni(II) (M. Wt. 517.12) and Zn(II) (M. Wt. 559.84) complexes, respectively (Additional file 1: Fig. S2). For instance, the Co(II) complex spectrum offered molecular ion peak at *m/z* = 519.19 (28.20%) which corresponding to the [M + 2] formula (M. Wt. 517.36). Moreover, the spectrum displayed a base peak at *m/z* = 483.39 (100.0%) which attributed to loss the two hydroxy groups giving C_24_H_16_CoN_4_O_4_^2**.**^ (F. Wt. = 483.35) that afterward underwent loss of furan ring giving a peak at *m/z* = 416.48 (36.63%) owing to the fragment C_20_H_13_CoN_4_O_3_ (F. Wt. = 416.28) (Fig. 3B). Further fragmentation steps were observed at *m/z* = 404.16, 287.32 and 259.99 which resulted from losing carbon, benzaldehyde and nitrogen fragments, respectively (Scheme 3).

### Electronic spectra and magnetic moments

**Fig. 4:**
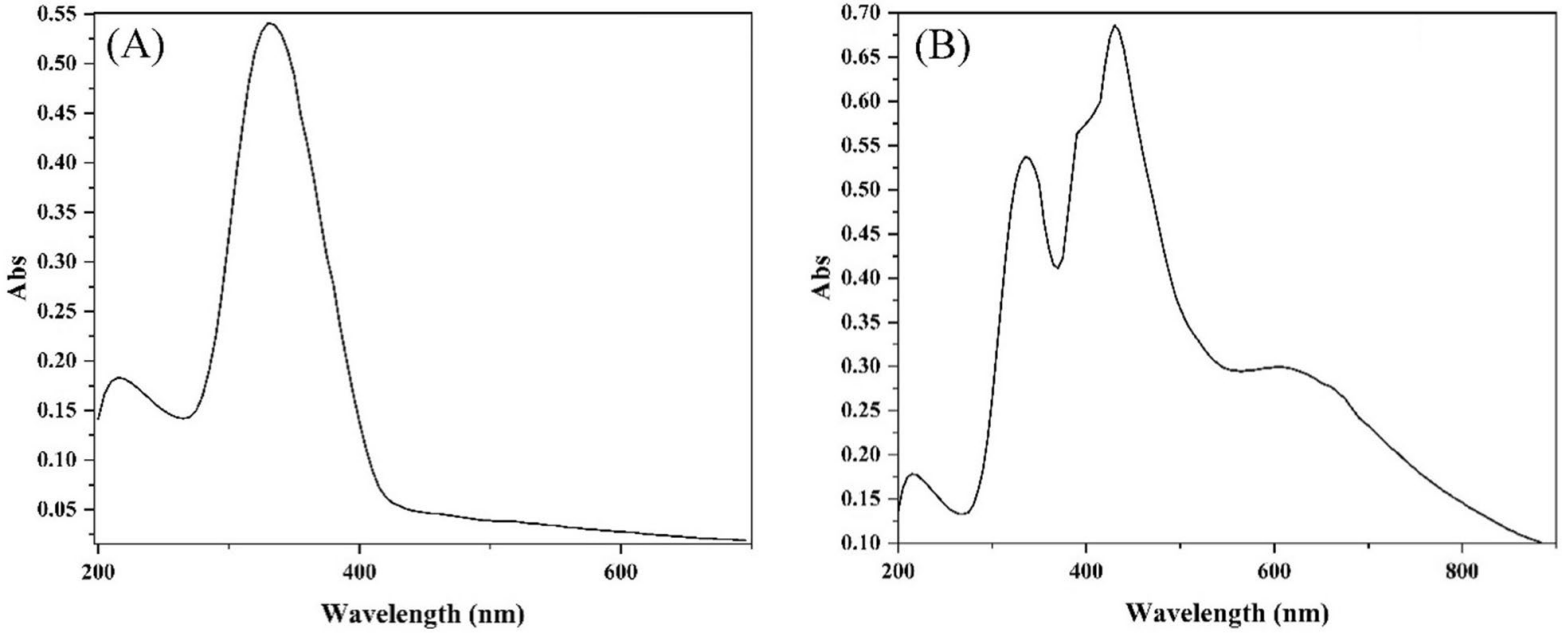
The electronic spectrum of the ligand (**A**) and Cu(II) complex (**B**)

The electronic spectrum of the H_2_L ligand, in DMSO, displayed two bands, the first observed at 46,510 cm^−1^ attributed to the π→π* transition of phenyl and furan rings while the second were at 30,300 cm^−1^ and assigned to π→π* transition of both carbonyl and azomethine groups [42]. In addition, a shoulder was observed at 21,740 cm^−1^ and ascribed to the n→π* transition of the carbonyl and azomethine groups [42] (Fig. 4A).

On comparison with ligand spectrum, the Cu(II) complex’s spectrum, in DMSO, displayed two bands at 46,510 and 29,850 cm^−1^ with a shoulder at 25,316 cm^−1^ were assigned to the intra-ligand transitions (π→π*)_Ar_, (π→π*)_C=X_ and (n→π*)_C=X_, respectively (Fig. 4B). Furthermore, the two new bands at 23,256 and 15,270 cm^−1^ were attributed to the ligand to metal charge transfer (LMCT) transition and ^2^T→^2^E transition of tetrahedral geometry [51, 52] (Table 3). In addition, the complex exhibited magnetic moment 2.17 B.M. that is in the normal range of Cu(II) complexes regardless of their stereochemistry [52], 1.75–2.20 B.M.

In DMSO, the spectrum of the Co(II) complex was carried out and showed two bands at 16,395 and 14,525 cm^−1^ attributed to ^4^T_1g_(F)→^4^T_1g_(P) (υ_3_) and ^4^T_1g_(F)→^4^A_2g_(P) (υ_2_) transitions, respectively, suggesting an octahedral geometry around the metal ion [51]. The band at 19,415 cm^−1^ was assigned to the ligand to metal charge transfer (LMCT) while the two bands observed at 25,315 and 22,990 cm^−1^ were corresponding to the n→π* transition of the azomethine groups (Additional file 1: Fig. S3). The spectral data were utilized to estimate the ligand field parameters, υ_1_, B and 10Dq, using the spin allowed transitions of the d^7^-system and were found to be 6790, 703, and 7730 cm^−1^, respectively, which were in the octahedral structure range [25, 51]. The magnetic moment of the Co(II) complex was found to be 5.16 B.M., in accordance with the usual values of octahedral geometry, 4.3–5.2 B.M. [53] (Table 4).

**Table 4:** Electronic spectra transitions and magnetic moment of the H_2_L and its complexes

Compound	Band position (cm^−1^) (transition)	µ_eff_ (B.M.)
H_2_L	46,510 (π→π*)_Ar_; 30,300 (π→π*)_C=X_; 21,740 (n→π*)_C=X_	–
Cu(II)	46,510 (π→π*)_Ar_, 29,850 (π→π*)_C=X_; 25,316 (n→π*)_C=X_; 23,256 (LMCT); 15,270 (^2^T→^2^E)	2.17
Co(II)	46,510 (π→π*)_Ar_; 29,850 (π→π*)_C=X_; 25,315 (n→π*)_C=X_; 22,990 (n→π*)_C=N*_; 19,415 (LMCT); 16,395 (^4^T_1g_(F)→^4^T_1g_(P) (υ_3_)); 14,525 (^4^T_1g_(F)→^4^A_2g_(P) (υ_2_))	5.16
Ni(II)	46,510 (π→π*)_Ar_; 29,850 (π→π*)_C=X_; 25,315 (n→π*)_C=X_; 19,610 (LMCT); 15,505 (^3^T_1_(F)→ ^3^T_1_(P) (ν_3_)	3.39
Zn(II)	46,510 (π→π*)_Ar_; 29,850 (π→π*)_C=X_; 25,315 (n→π*)_C=X_; 23,256 (n→π*)_C=N*_; 19,610 (LMCT)	–

Ni(II) complex spectrum presented a sharp band at 19,610 cm^−1^ in addition to a broad band at 15,505 cm^−1^ which assigned to LMCT and ^3^T_1_(F)→^3^T_1_(P) (ν_3_) transitions, respectively, of tetrahedral geometry around Ni(II) ions [51]. Furthermore, the magnetic moment values of the complexes were found to be 3.39 B.M., in accordance with the standard values of the tetrahedral geometry (3.2–4.1 B.M.) [51]. Finally, the Zn(II) complex displayed three bands at 46,510, 29,850 and 25,315 cm^−1^ along with two new bands at 23,256 and 19,610 cm^−1^ attributed to intra-ligand transitions, (π→π*)_Ar_, (π→π*)_C=X_, (n→π*)_C=X_, (n→π*)_C=N*_ [42] and LMCT [51, 52], respectively (Additional file 1: Fig. S3).

### ESR spectra of Cu(II) complex

**Fig. 5:**
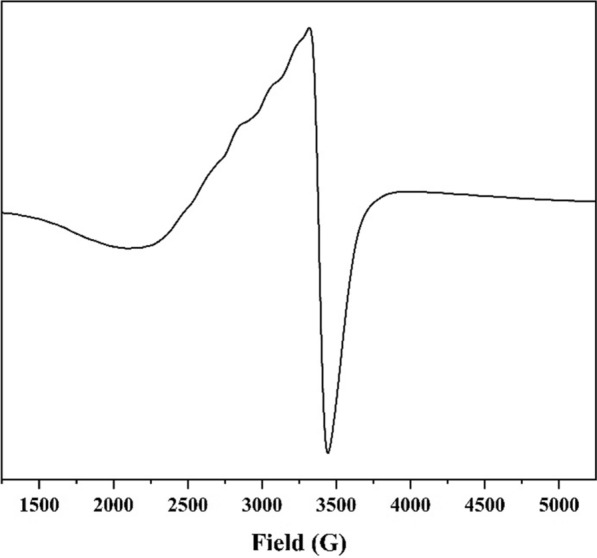
The ESR spectrum of Cu(II) complex

The ESR spectrum of Cu(II) complexes, that has tetragonal or distorted octahedral geometry, displayed g-tensor values g_||_ > g_⊥_ > 2.0023, which indicated that their ground state is [54]. In accordance, the solid-state spectrum of the Cu(II) complex presented g-tensor values of g_||_ = 2.14 and g_⊥_ = 2.05which cleared out that the Cu(II) has a ground state with significant covalent nature of metal–ligand bonds as its g_||_ < 2.3 [55] (Fig. 5). If the value of the axial symmetry parameter G, who is defined as (g_||_ − 2)/(g_⊥_ − 2), is less than 4, it indicates that the exchange interaction was reasonable and the local tetragonal axes were misaligned [56]. Consequently, the G value of the present Cu(II) complex was ascertained to be 3.10.

Furthermore, the decrease of A_||_ with increasing g_||_ is an evidence of increasing the tetrahedral distortion within the Cu(II) coordination sphere [52, 57]. To determine the distortion degree, the empirical index, g_||_/A_||_ factor, was calculated, where the square planar complexes revealed value in 105–135 range, while the distorted tetrahedral exhibited larger values [52, 58]. The spectrum displayed four hyperfine lines endorsing the monomeric nature of the Cu(II) complex (A_||_ = 155 × 10^−4^ cm^−1^). Hence, the g_||_/A_||_ factor has been calculated to be 138, which implied the presence of significant dihedral angle distortion in the xy-plane and tetrahedral distortion from square planar geometry [52].

As a measure of covalency, the orbital reduction factor, K, was determined using the following expressions (1–3) [59, 60], where K = 1 for ionic, K < 1 for covalent environments, ${K}_{\parallel }$ and ${K}_{\perp}$ are the parallel and perpendicular components of orbital reduction factor, respectively.

1 $${\text{K}}_{\parallel}^2 = \frac{{\left( {{{\text{g}}_{\parallel}} - 2.0023} \right)}}{{8 \times {\lambda_\circ}}} \times {\text{d}}{-}{\text{d}}\;{\text{transition}}$$

2 $${\text{K}}_{\perp}^{2} = \frac{{\left({{\text{g}_{\perp}} - 2.0023}\right)}}{{2 \times {{\lambda}_\circ}}} \times {\text{d}}{-}{\text{d}}\;{\text{transition}}$$

3 $${{\text{K}}^2} = \frac{{\left( {{\text{K}}_{\parallel}^{2} + 2{\text{K}}_{\perp}^{2}} \right)}}{3}$$

The results showed that the complex had K = 0.63, ${K}_{\parallel}={0.57}$ and ${K}_{\perp}=0.65$, indicting the strong ionic character and in-plane π-bonding as ${{K}_{\parallel}} < {{K}_{\perp}}.$  

### Thermal analyses

**Fig. 6:**
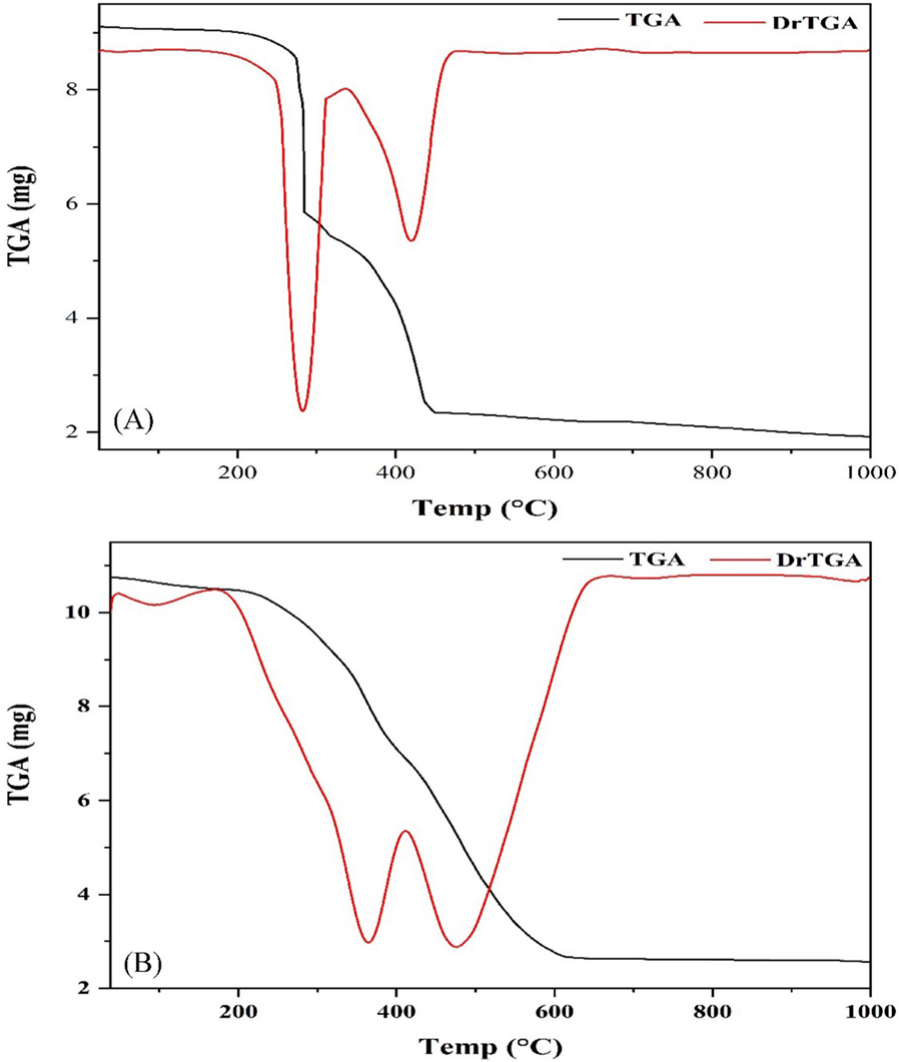
The TG curves of Cu(II) (**A**) and Zn(II) (**B**) complexes

To explore the isolated solid complexes thermal stability and aid in characterization of their chemical structures, thermal gravimetric analysis (TGA) of was carried out. The thermogram of the Cu(II) complex displayed the first degradation step at 165–335 °C region and was attributed to loss of hydroxy phenyl fragments (C_14_H_10_O_2_) (Found 40.70; Calcd. 40.28%). The subsequent stage was observed at 335–475 °C and assigned to removal of furan rings along with other fragments, C_10_H_8_NO_2_, (Found 33.70; Calcd. 33.37%). The third step has been extended from 475 to 1000 °C and ascribed to loss of nitrogen molecule (Found 4.97; Calcd. 5.37%) which led to a residue of CuNO_2_ (Found 20.63; Calcd. 20.99%) (Fig. 6).

Likewise, the TG curve of the Co(II) complex exhibited three decomposition stages, the first stage at 180–330 °C corresponding to loss of hydroxy phenyl in addition to hydroxyl moieties (Found 21.86; Calcd. 21.28%) whereas the second was observed in 330–482 °C range and assigned to degradation of the ligand through losing C_18_H_12_NO_2_ (Found 52.90; Calcd. 53.03%) (Additional file 1: Fig. S4). Eventually, the 3rd stage was spread over the 482–1000 °C region and accompanied with losing of nitrogen molecule to result in residue of CoNO_2_ (Found 20.15; Calcd. 20.28%) (Table 5).

**Table 5:** TGA of the isolated metal complexes

Complex	Temp. range (°C)	Wt. loss, %	Fragment	Fragment, %
Cu(II)	165–335	40.70	2HOPhC (C_14_H_10_O_2_)	40.28
	335–475	33.70	2furanC + N (C_10_H_8_NO_2_)	33.37
	475–1000	4.97	N_2_	5.37
	Residue	20.63	CuNO_2_	20.99
Co(II)	180–330	21.86	PhOH + OH (C_6_H_6_O_2_)	21.28
	330–482	52.90	Lig. Dec. (C_18_H_12_NO_2_)	53.03
	482 − 100	5.09	N_2_	5.41
	Residue	20.15	CoNO_2_	20.28
Ni(II)	185–375	46.25	2HOPhCN (C_14_H_10_N_2_O_2_)	46.07
	375–1000	39.28	2furCN + O (C_10_H_8_N_2_O_3_)	39.49
	Residue	14.47	NiO	14.44
Zn(II)	40–170	3.31	H_2_O	3.33
	170–413	34.03	2PhOH (C_12_H_10_O_2_)	34.37
	413–1000	39.84	2FurCNN (C_10_H_8_N_4_O_2_)	39.90
	Residue	22.82	Zn(CO)_2_	22.40

Alternatively, the Ni(II) complex graph showed only two broad degradation stages, the 1st was started at 185 to 375 °C and attributed to loss of hydroxy cyanophenyl fragments, C_14_H_10_N_2_O_2_, (Found 46.25; Calcd. 46.07%) (Additional file 1: Fig. S4). While, the 2nd was observed at 375–1000 °C and assigned to complete decomposition of the ligand resulting in a residue of NiO (Found 14.47; Calcd. 14.44%) (Table 5).

Finally, the TG curve of the Zn(II) complex presented the first degradation earlier than other complexes, 40–170 °C, where it was assigned to loss of the outside coordination sphere water molecule (Fig. 6). The second step was displayed at 170–413 °C region and attributed to loss of hydroxy phenyl fragments, C_12_H_10_O_2_, (Found 34.03; Calcd. 34.37%) whereas the third one was observed at 413–1000 °C range due to decomposition of the ligand to give a residue of Zn(CO)_2_ (Found 22.82; Calcd. 22.40%) (Table 5).

### DFT computations

The DFT calculations were carried out to obtain the geometrically optimized structure in addition to the frontier molecular orbitals shapes and energy of the ligand and its metal complexes. The resulting geometrical parameters, bond length, angle and dihedral angle, were compared with those obtained from x-ray single crystal of analogous molecules [61, 62] where small difference was observed. The difference may be ascribed to that the theoretical calculations were carried out on single molecule in gaseous state, where no intermolecular columbic interactions, whereas, the experimental were acquired for molecules interacting in solid crystal lattice [63].

**Fig. 7:**
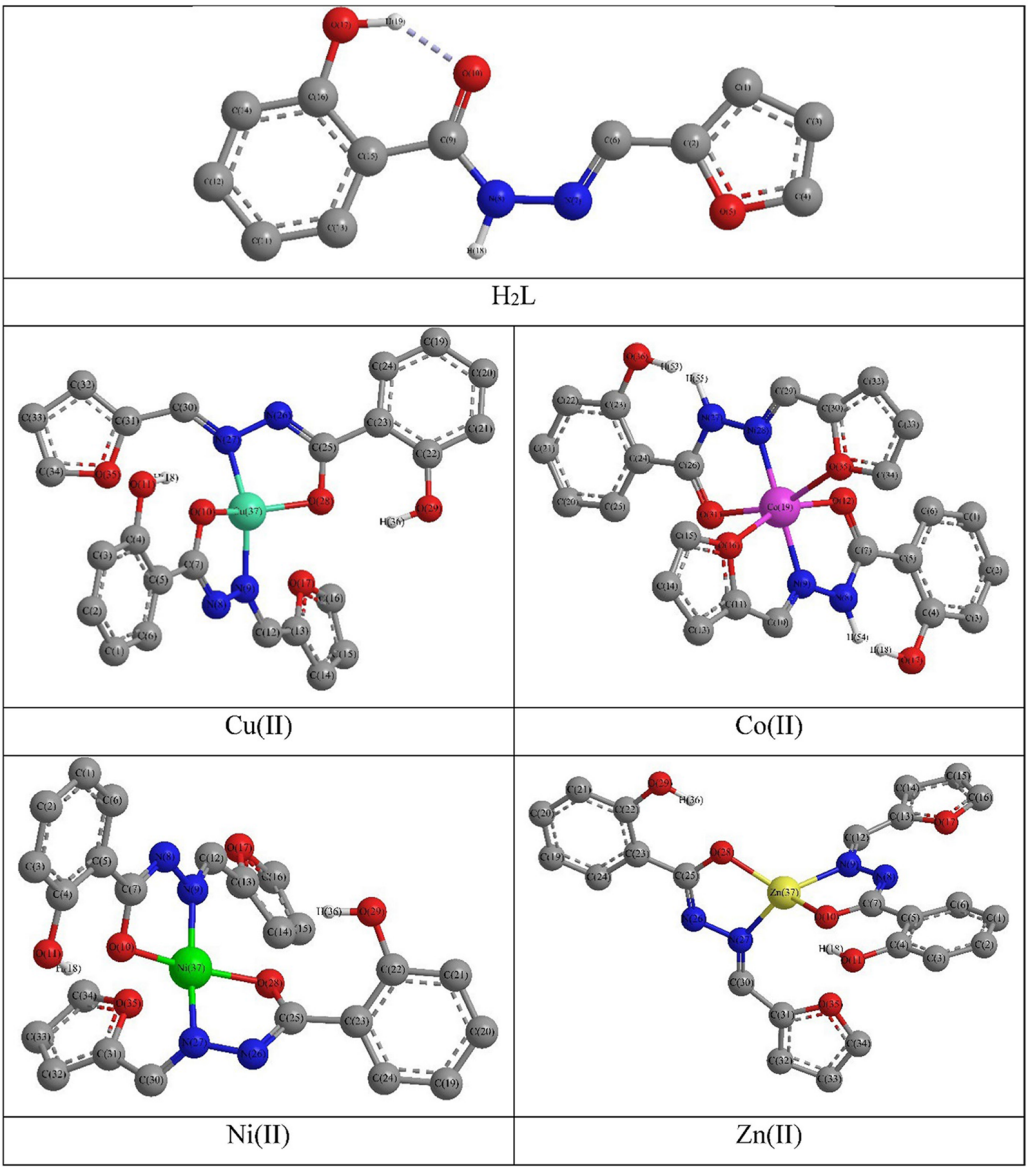
The optimized structures of H_2_L and it metal complexes

H_2_L optimized structure disclosed that it has a planar structure in which the hydroxyl and carbonyl group were alongside but the hydroxyl’s hydrogen atom was orient away from the carbonyl’s oxygen, consequently, there are no H-bond (Additional file 1: Table S1). Meanwhile, the optimized structures of the metal complexes revealed that all have distorted tetrahedral except the Co(II) which has an octahedral stereochemistry in accordance with the suggested configurations (Fig. 7).

The comparison of geometrical parameters data of ligand with those of complexes reveal that:

i) The ligand C^2^_(fur)_–O^1^_(fur)_ bond length was 1.40 Å and did not alter on coordination of the furfural oxygen to Co(II) ion (Additional file 1: Table S2).
ii) In H_2_L, the N_(imin)_–NH_(Hz)_ and CO_(sal)_–OC_(sal)_ bonds were 1.39 and 1.28 Å, while due their involvement of the N_(imin)_ and OC_(sal)_ in chelation to the metal ions and enolization, their lengths became in 1.27–1.40 and 1.22–1.35 Å range, respectively.
iii) Generally, the metal complexes have distorted stereochemistries as indicated from the bond length data, i.e., M–N_(imin)_, 1.84–2.03 Å, was longer than M–OC_(sal)_, 1.80–1.95Å.
iv) Moreover, the bond angles data presented another distortion in the geometrical configuration, e.g. in octahedral Co(II), the angles were deviate from the standard values, 90° and 180° by 2.0° to 11.0° (Additional file 1: Table S3).

Additionally, the highest occupied and lowest unoccupied molecular orbitals (HOMO and LUMO) govern how the molecule interact with other species where HOMO acts as electron donor while LUMO acts as electron acceptor [10, 33]. Thus, the higher the HOMO energy, the easier to donate electrons whereas LUMO accepts electrons easier when it has low energy [10, 33]. The LUMO-HOMO energy gap reflects the chemical activity of the molecule, where a molecule with a small energy gap is soft molecule, more polar, chemically reactive and less kinetically stable [64]. Also, the low HOMO-LUMO gap indicates facile charge transfer interaction taking place within the molecule [63].

**Fig. 8:**
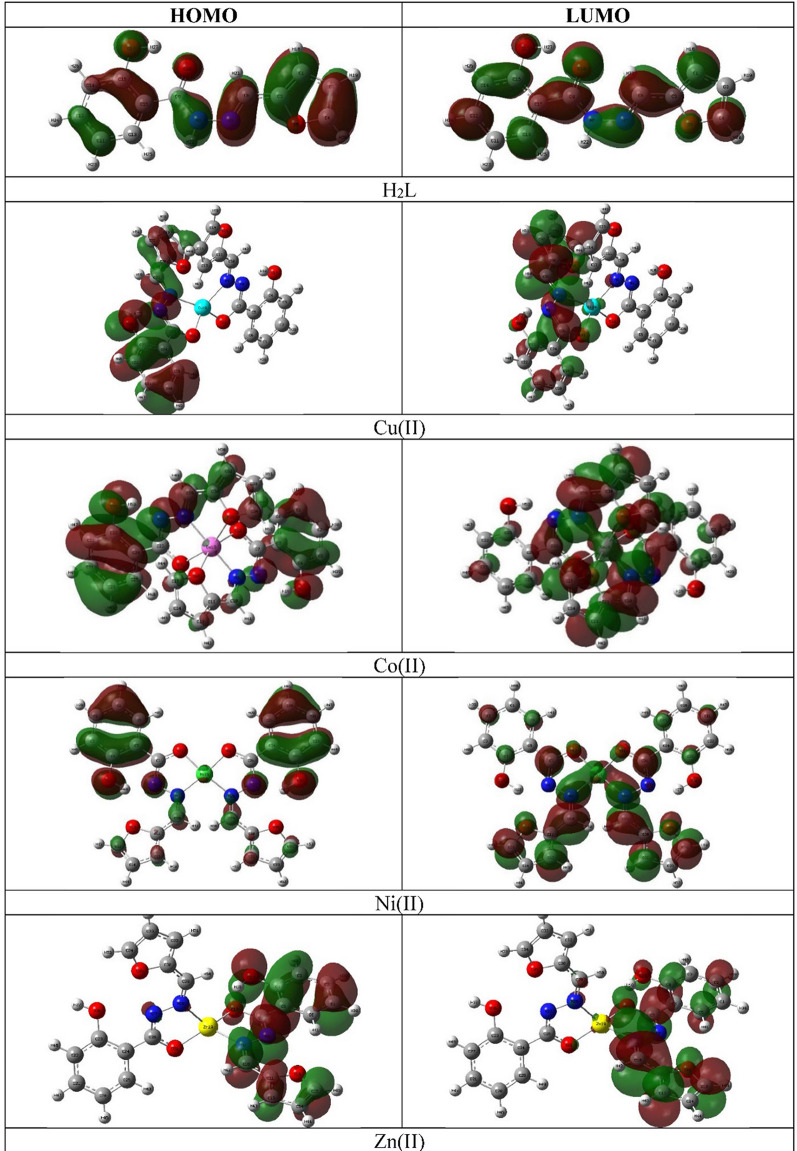
3D plots of FMO’s for H_2_L and its complexes

The LUMO-HOMO plots for the ligand showed that the HOMO was mainly consisted of the non-bonding orbitals, that occupied by lone pair of electrons, of the oxygen and nitrogen atoms in addition to the π-orbitals of whole molecule while the LUMO was formed from the π*-orbitals of the whole molecule. Thus, the HOMO-LUMO charge transfer may be described as π→π* and n→π* transitions. On the other hand, the octahedral metal complexes plot showed that their HOMO was mainly made of the π-orbital of the whole molecule and heteroatoms lone pair of electrons in addition to minor contribution of the metal ion. Whereas, their LUMO was constructed from the π*-orbital of the molecule with contribution of the central metal ion. On contrary, the square planar Ni(II) complex has different FMO’s configurations where the HOMO was built of the π-orbital of the phenyl ring and lone pair of electrons with minor involvement of the Ni(II) ion while the LUMO was made of the π*-orbital of the furfural ring with more contribution of the metal (Fig. 8).

According the HOMO-LUMO energy data, the ligand has the highest HOMO energy, E_H_ = − 6.32 eV, revealing its electron donation character while the metal complexes displayed close values, − 5.71 to − 6.06 eV. Moreover, the LUMO data indicated that the Ni(II) complex has the highest energy value, E_L_ = − 2.84 eV. The calculated HOMO-LUMO energy gap, ΔE_H−L_, of the ligand was the highest value, 4.05 eV, while, Ni(II) complex has the lowest, 3.07 eV (Table 6).

**Table 6:** The FMO’s energies and chemical reactivity descriptors of the ligand and its complexes (eV)

Compound	E_H_	E_L_	ΔE_H−L_	χ	η	S	ω	ω+	ω−
H_2_L	− 6.32	− 2.27	4.05	4.29	2.02	0.49	4.56	2.66	6.96
Cu(II)	− 6.06	− 2.83	3.23	4.45	1.61	0.62	6.13	4.11	8.55
Co(II)	− 5.83	− 2.58	3.25	4.21	1.62	0.62	5.45	3.55	7.76
Ni(II)	− 5.91	− 2.84	3.07	4.37	1.54	0.65	6.23	4.23	8.61
Zn(II)	− 5.71	− 2.41	3.29	4.06	1.65	0.61	5.01	3.18	7.25

Finally, the E_H_ and E_L_ were used to estimate other chemical reactivity descriptors such as electronegativity (χ), which illustrate that molecule behaves has Lewis’s acidic or basic character, and global hardness (*η*) which measure the charge transfer resistance [65]. Moreover, the global softness (*δ*), describes the molecule receiving electrons capacity, and electrophilicity (*ω*) that measures of energy reduction due to HOMO-LUMO electron flow between, were calculated as follows, (4–9) [65]:

4 $${\chi} = - \frac{1}{2}\left({{E_{HOMO}} + {E_{LUMO}}}\right)$$

5 $${\eta} = - \frac{1}{2}\left({{E_{HOMO}} - {E_{LUMO}}}\right)$$

6 $$\delta =\frac{1}{\eta}$$

7 $${\omega} = \frac{{{\chi^2}}}{{8\eta}}$$

8 $${\omega^{-}} = \frac{{{{\left({3I+A}\right)}^2}}}{{16\left({I-A}\right)}}$$

9 $${\omega^{+}} = \frac{{{{\left({I+3A} \right)}^2}}}{{16\left({I-A}\right)}}$$

As shown in Table 6, the ligand displayed the highest global hardness, 2.02 eV, whereas the Ni(II) complex had the lowest value, 1.54 eV. On contrary, the softness presented reversed order where Ni(II) complex has the highest softness, 0.65 eV. The molecules with electrophilicity index (ω) > 1.5 eV, that measure of acquiring extra electronic charge from the environment stabilization energy, were considered as strong electrophile [66, 67]. Therefore, the studied compounds were strong electrophile as they exhibited ω index ranged from 4.56 to 6.23 eV following the order L < Zn(II) < Co(II) < Cu(II) < Ni(II). Likewise, the electron donating (ω^+^) and acceptance (ω^−^) powers data, which demonstrated the capability to give and receive electrons, respectively, obeyed the pervious order but they exhibited more donation tendency, 2.66–3.18 eV, than acceptance, 6.96–7.25 eV, where smaller values signify enhanced transaction [66, 67] (Table 6).

### Cytotoxicity activity

**Fig. 9:**
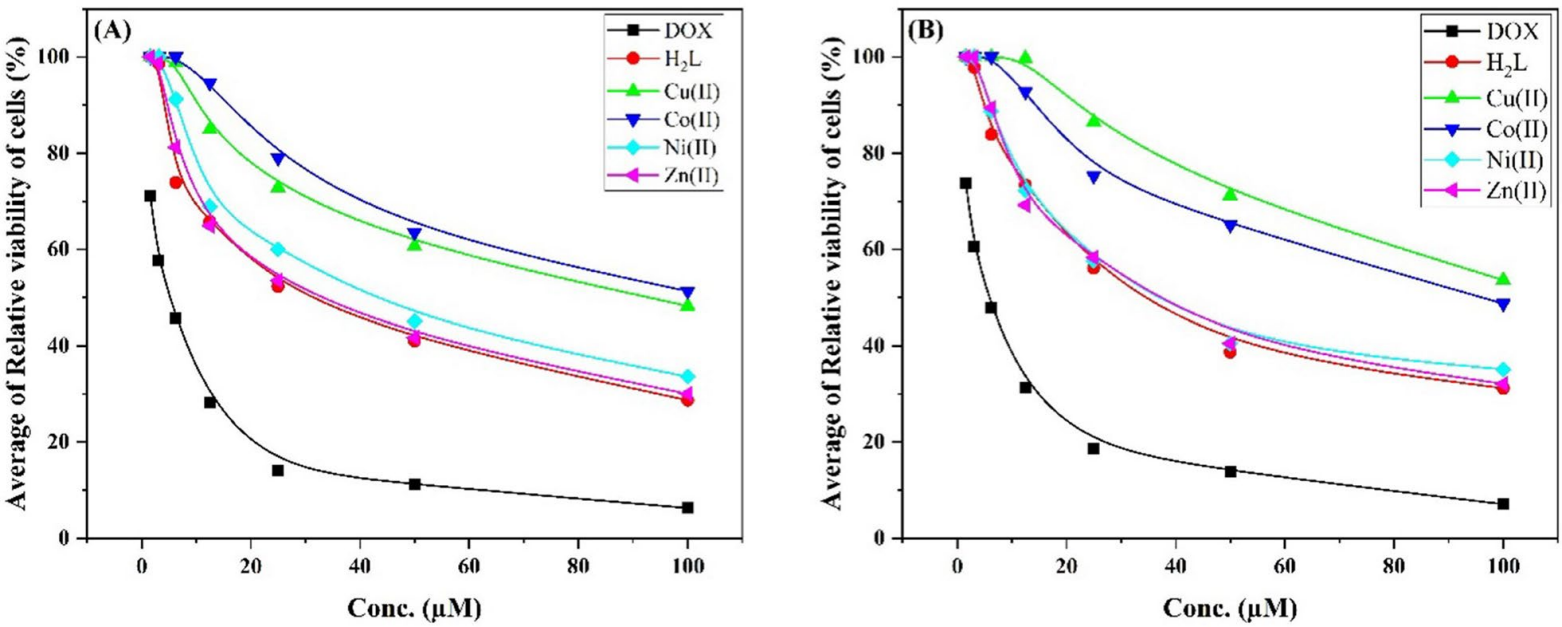
The cell viability (%) of the ligand and its complexes against HePG-2 (**A**) and HCT-116 (**B**)

The cytotoxicity of the ligand and its metal complexes has been evaluated in vitro against two cell lines, HePG-2 and HCT-116, which represent liver and colon cancer, respectively. Doxorubicin was used as a reference medication for comparison, and the results are shown in Table 7. The results revealed that the ligand exhibited moderate cytotoxicity towards HePG-2 and HCT-116 (IC_50_ = 30.72 and 35.40 µM). Similarly, both of the Ni(II) and Zn(II) complexes exhibited moderate cytotoxicity (IC_50_ = 32.83–41.47 µM). However, the Cu(II) and Co(II) complexes displayed weak impacts on the examined cell lines. It is noteworthy that the cytotoxicity of H_2_L was comparatively higher than that of metal complexes which may be correlated to the presence of several free active sites in H_2_L which facilitate binding to the protein. But, after complexation, some of the active sites no longer available due to their involvement in metal ion chelation. Furthermore, the metal complexes have large size, since their stoichiometric ratio was 1:2 (M:L), which may hinder their ability to penetrate through the cell membrane and hence reduce the cytotoxic effects. Despite the value of IC_50_, the Zn(II) complex was the most potent against the two cell lines. The average cells relative viability percent for both cell lines presented that the ligand had the least viability and then the Zn(II) complex (Fig. 9).

**Table 7:** In vitro cytotoxic activity IC_50_ of the ligand and its complexes

Compound	In vitro cytotoxicity IC_50_ (µM)*	
	HePG-2	HCT-116
Doxorubicin	4.50 ± 0.2	5.23 ± 0.3
H_2_L	30.72 ± 2.2	35.40 ± 2.2
Cu(II)	82.63 ± 4.2	> 100
Co(II)	93.56 ± 4.7	89.01 ± 4.5
Ni(II)	41.47 ± 2.6	39.19 ± 2.4
Zn(II)	32.83 ± 2.3	37.27 ± 2.4

*IC_50_ (µM): 1–10 (very strong). 11–20 (strong). 21–50 (moderate). 51–100 (weak) and above 100 (non-cytotoxic)

## Conclusion

The spectral analyses of *N′*-(furan-2-ylmethylene)-2-hydroxybenzohydrazideligand revealed its existence in the keto-form. While, upon reaction with the metal acetate, the resulting metal complexes have a 1:2 (M:L) stiochoimetry and coordinated in enol-form as mononegative bidentate via enolic O and azomethine N, however, in Co(II) complex, the ligand was reacted in mononegative tridentate manner through enolic and furan O in addition to azomethine N. The energy gap of FMO’s, determined by DFT computations, showed that the ligand and Ni(II) complex have the highest and lowest values, respectively, following the order H_2_L > Zn(II) > Co(II) > Cu(II) > Ni(II). The cytotoxicity activity against two cell lines, HePG-2 and HCT-116, of H_2_L was relatively higher (lower IC_50_) than that of metal complexes, H_2_L < Zn(II) < Ni(II) > Cu(II) < Co(II) which may be correlated to blocking of some active sites in H_2_L that bonded to the metal ions.

### Supplementary Information

**Additional file 1: Figure S1.** IR spectra of Cu(II), Ni(II) and Zn(II) complexes. **Figure S2.** The mass spectra of Cu(II) (A), Ni(II) (B) and Zn(II) (C) complexes. **Figure S3.** The electronic spectra of Co(II) (A), Ni(II) (B) and Zn(II) (C) complexes. **Figure S4.** The TG curves of Co(II) (A) and Ni(II) (B) complexes. **Table S1.** Bond length data of the ligand and its complexes. **Table S2.** Bond angle data of the ligand and its complexes. Table S3. Dihedral angle data of the ligand and its complexes.

## Data Availability

The datasets used and/or analyzed during the current study are available from the corresponding author on reasonable request.
