# Supplementary material for: N′-(Furan-2-ylmethylene)-2-hydroxybenzohydrazide and its metal complexes: synthesis, spectroscopic investigations, DFT calculations and cytotoxicity profiling
Source: BMC Chem. 2024 Jan 28;18(1):22. doi: 10.1186/s13065-023-01098-8 (PMC10823611; doi:10.1186/s13065-023-01098-8)
Supplement: Supplementary file 1 — Additional file 1: Figure S1. IR spectra of Cu(II), Ni(II) and Zn(II) complexes. Figure S2. The mass spectra of Cu(II) (A), Ni(II) (B) and Zn(II) (C) complexes. Figure S3. The electronic spectra of Co(II) (A), Ni(II) (B) and Zn(II) (C) complexes. Figure S4. The TG curves of Co(II) (A) and Ni(II) (B) complexes. Table S1. Bond length data of the ligand and its complexes. Table S2. Bond angle data of the ligand and its complexes. Table S3. Dihedral angle data of the ligand and its complexes. [file 13065_2023_1098_MOESM1_ESM.doc]

| **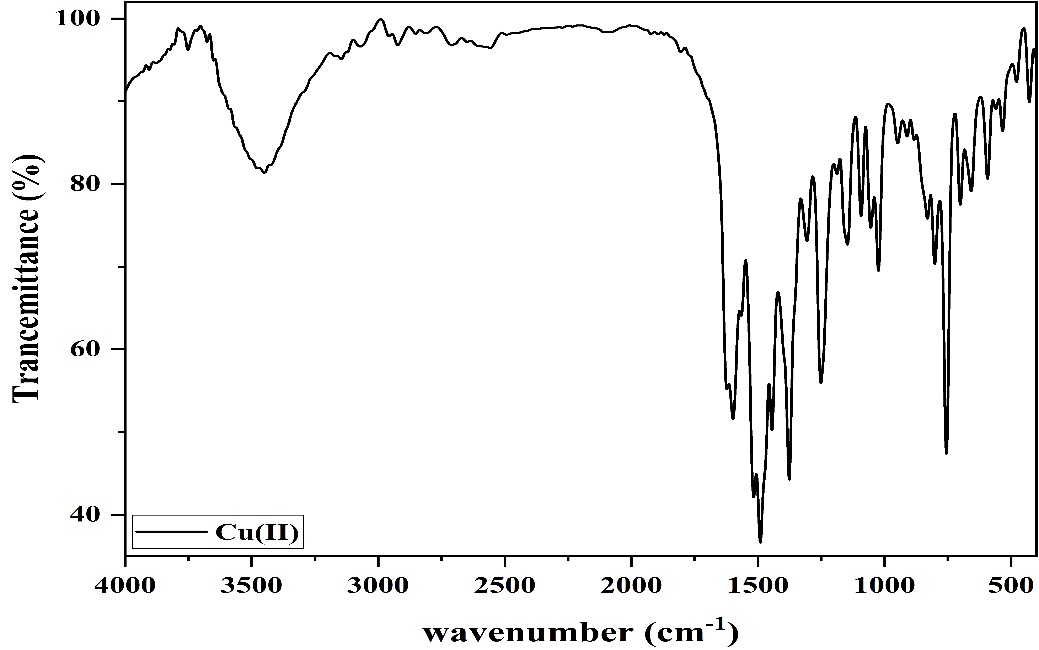** |
| --- |
| **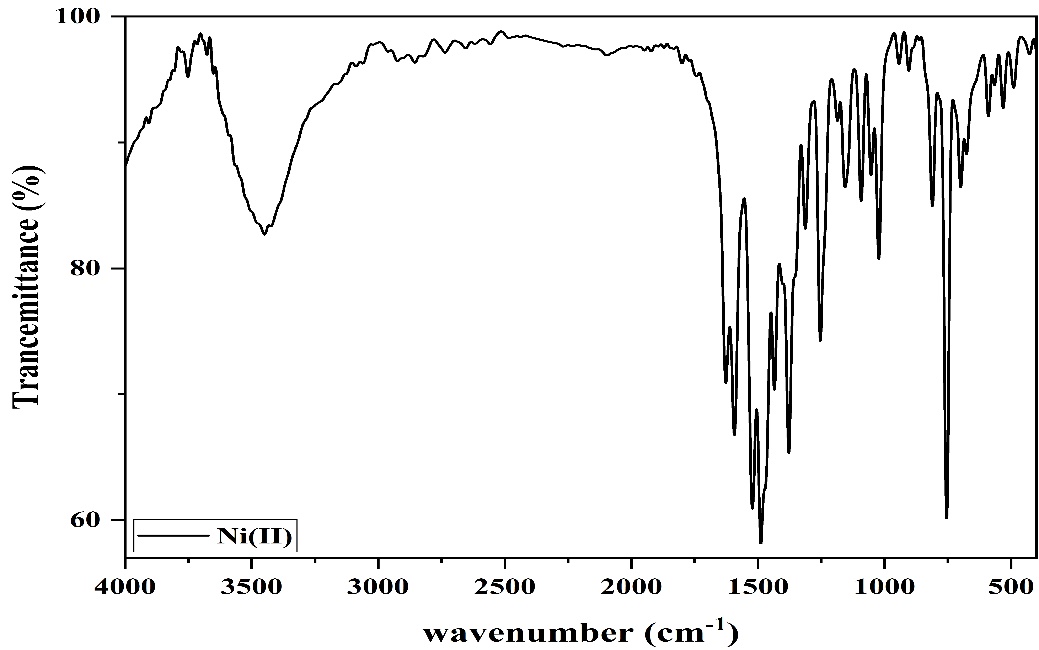** |
| **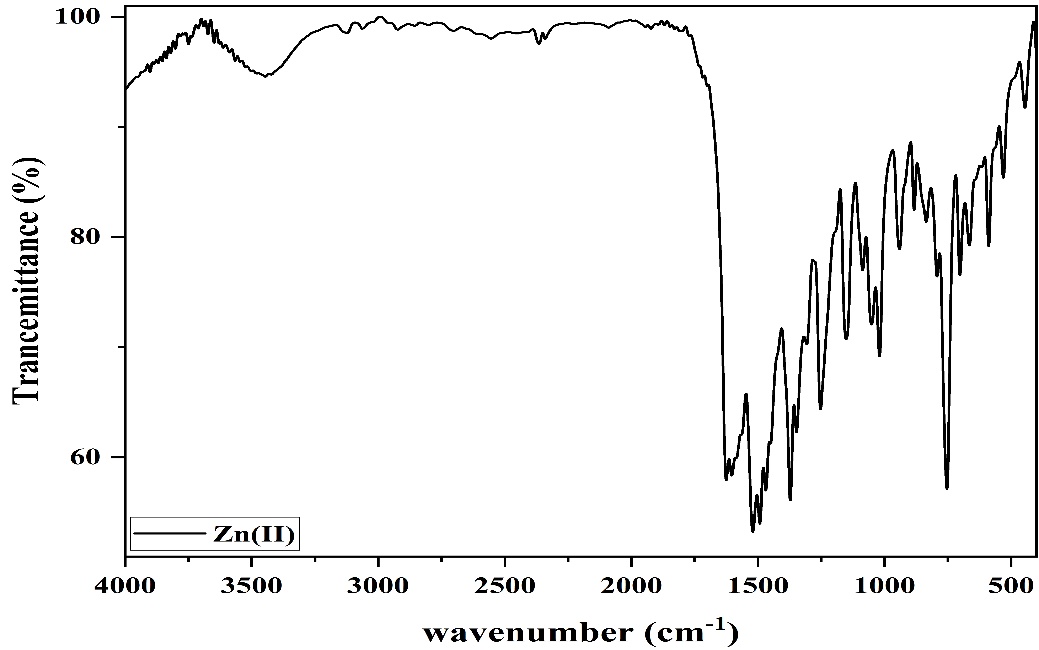** |

**Figure S1**. IR spectra of Cu(II), Ni(II) and Zn(II) complexes

| (A) | 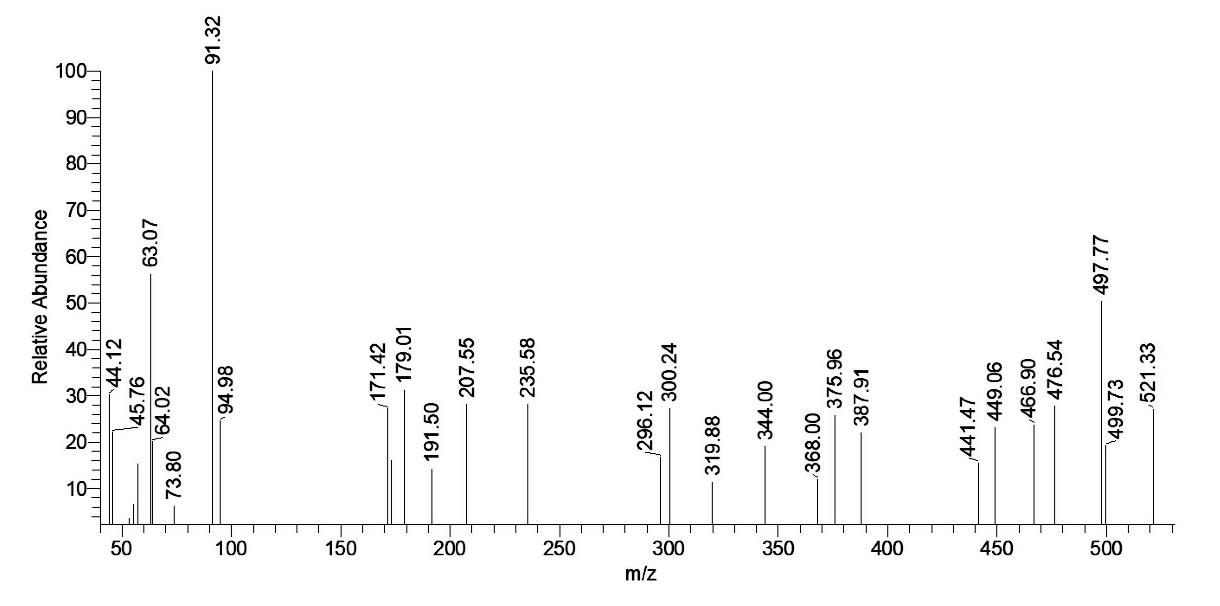 |
| --- | --- |
| (B) | 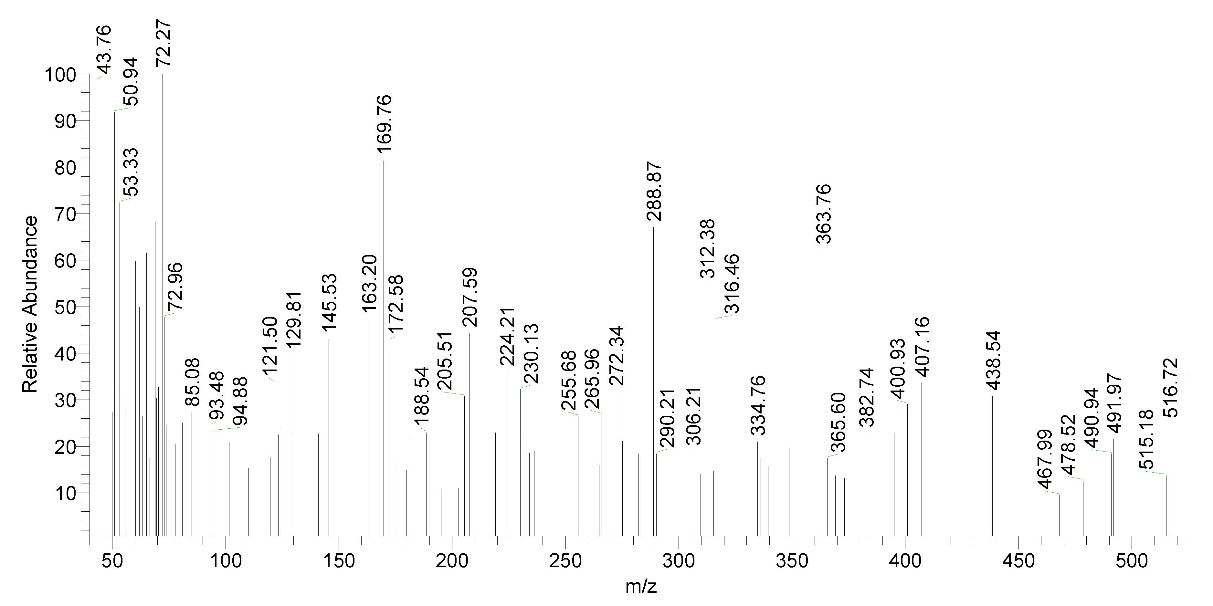 |
| (C) | 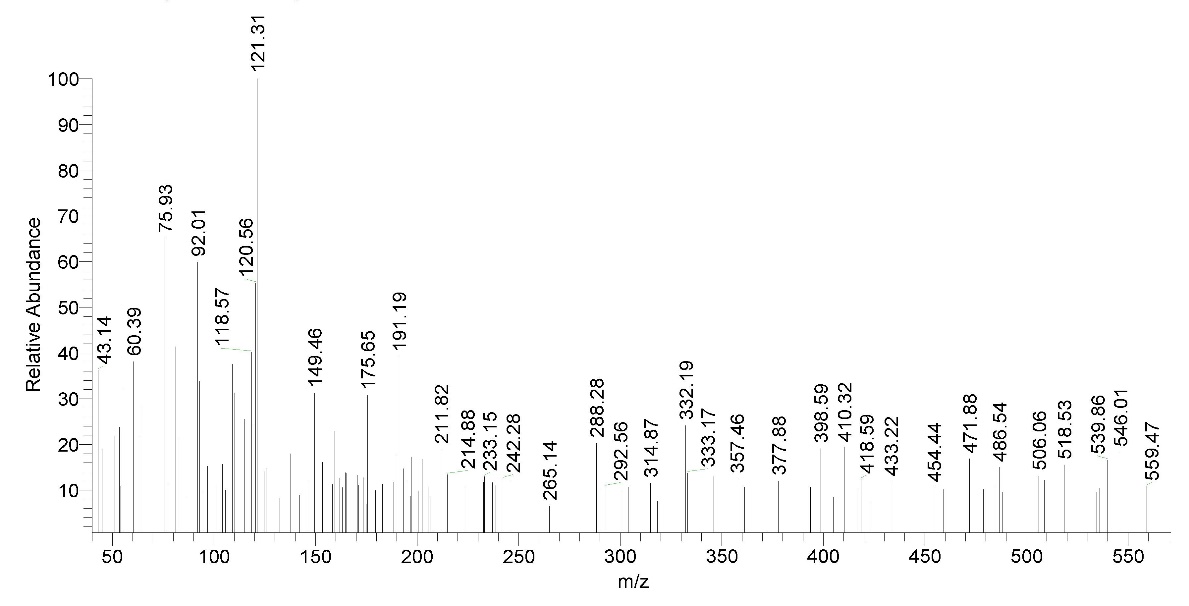 |

**Figure S2**. The mass spectra of Cu(II) (A), Ni(II) (B) and Zn(II) (C) complexes

| 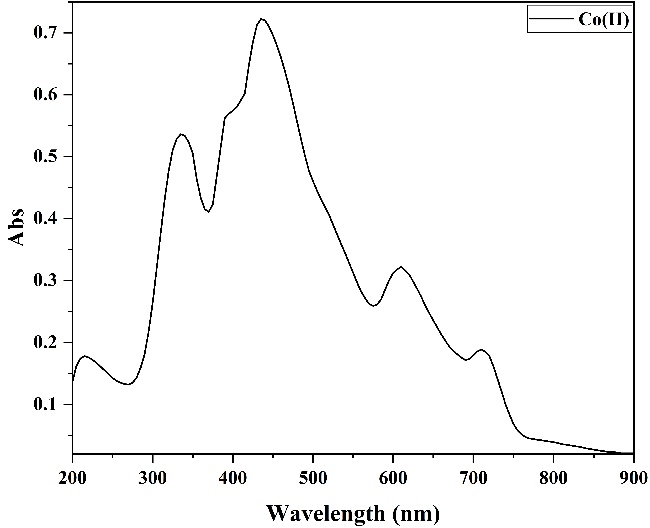 | 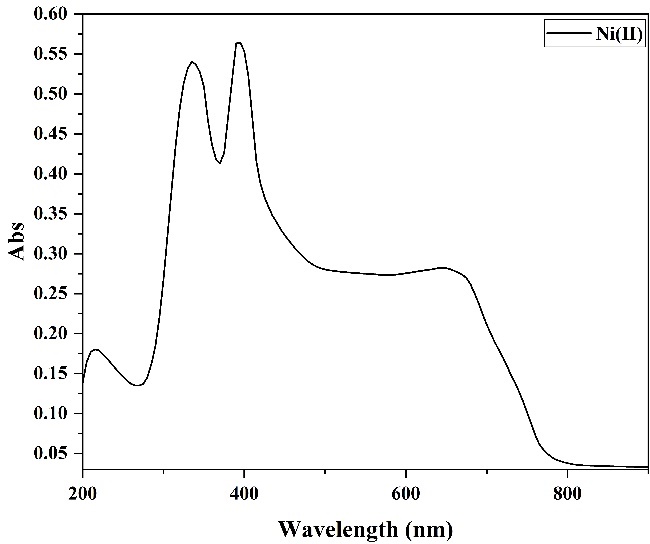 |
| --- | --- |
| (A) | (B) |
| 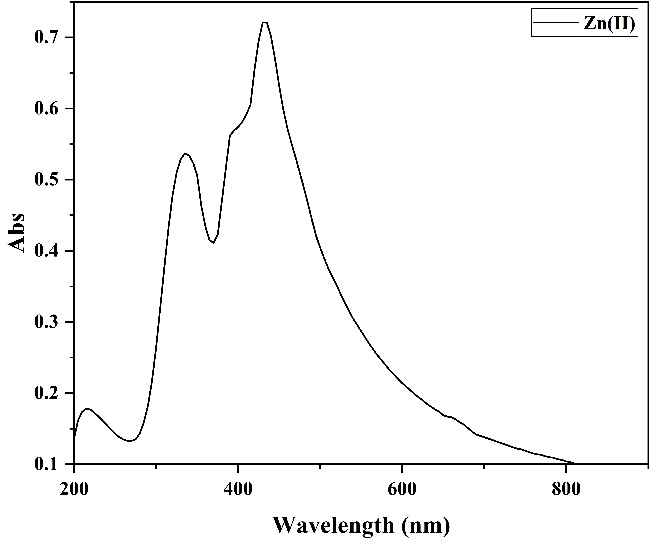 | |
| (C) | |

**Figure S3**. The electronic spectra of Co(II) (A), Ni(II) (B) and Zn(II) (C) complexes

| (A) | 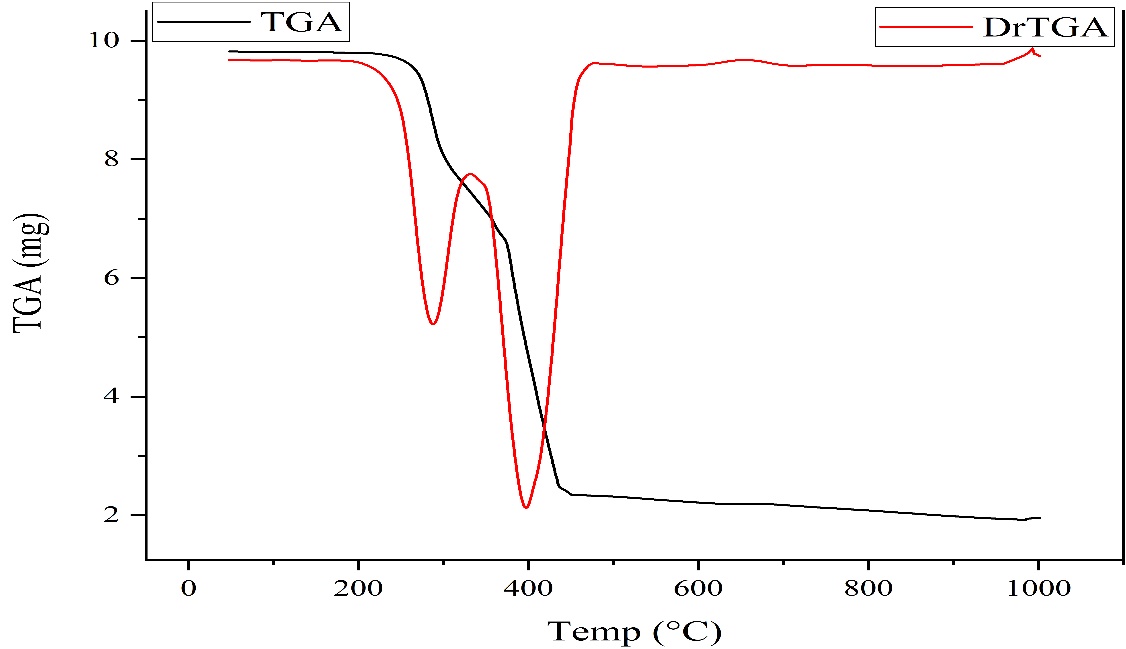 |
| --- | --- |
| (B) | 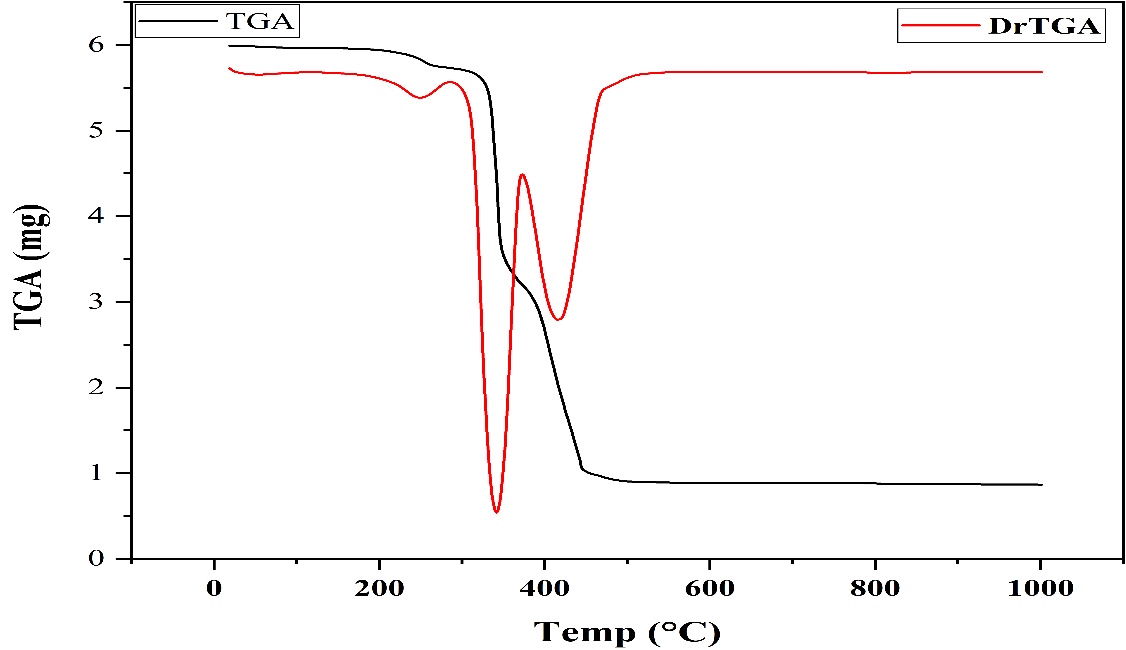 |

**Figure S4**. The TG curves of Co(II) (A) and Ni(II) (B) complexes.

**Table S1**. Bond length data of the ligand and its complexes

| **Ligand** | | Cu(II) | | Ni(II) | | Zn(II) | | Co(II) | |
| --- | --- | --- | --- | --- | --- | --- | --- | --- | --- |
| O(17)-H(27) | 1.00 | O(36)-H(37) | 1.02 | O(36)-H(37) | 1.00 | N(27)-H(54) | 1.01 | O(36)-H(53) | 0.97 |
| C(16)-O(17) | 1.37 | C(34)-O(35) | 1.39 | C(34)-H(53) | 1.10 | O(36)-H(37) | 0.98 | C(34)-H(52) | 1.09 |
| C(15)-C(16) | 1.42 | C(33)-C(34) | 1.38 | C(34)-O(35) | 1.23 | C(34)-O(35) | 1.39 | C(34)-O(35) | 1.40 |
| C(14)-C(16) | 1.40 | C(32)-C(33) | 1.44 | C(33)-H(52) | 1.10 | C(33)-C(34) | 1.38 | C(33)-H(51) | 1.09 |
| C(13)-C(15) | 1.41 | C(30)-O(35) | 1.40 | C(33)-C(34) | 1.34 | C(32)-C(33) | 1.43 | C(33)-C(34) | 1.38 |
| C(12)-C(14) | 1.39 | C(30)-C(32) | 1.39 | C(32)-H(51) | 1.10 | C(30)-O(35) | 1.40 | C(32)-H(50) | 1.09 |
| C(11)-C(13) | 1.39 | C(29)-C(30) | 1.43 | C(32)-C(33) | 1.34 | C(30)-C(32) | 1.40 | C(32)-C(33) | 1.43 |
| C(11)-C(12) | 1.41 | N(28)-C(29) | 1.31 | C(30)-O(35) | 1.23 | C(29)-C(30) | 1.44 | C(30)-O(35) | 1.41 |
| C(9)-C(15) | 1.48 | N(27)-N(28) | 1.40 | C(30)-C(32) | 1.34 | N(28)-C(29) | 1.31 | C(30)-C(32) | 1.39 |
| C(9)-O(10) | 1.28 | C(26)-O(31) | 1.32 | C(29)-H(50) | 1.10 | N(27)-N(28) | 1.41 | C(29)-H(49) | 1.10 |
| N(8)-H(22) | 1.00 | C(26)-N(27) | 1.36 | C(29)-C(30) | 1.34 | C(26)-O(31) | 1.34 | C(29)-C(30) | 1.44 |
| N(8)-C(9) | 1.37 | C(24)-C(26) | 1.47 | N(28)-C(29) | 1.27 | C(26)-N(27) | 1.35 | N(28)-C(29) | 1.32 |
| N(7)-N(8) | 1.39 | C(24)-C(25) | 1.42 | N(27)-H(37) | 1.04 | C(24)-C(26) | 1.49 | N(27)-N(28) | 1.35 |
| C(6)-N(7) | 1.30 | C(23)-O(36) | 1.38 | N(27)-N(28) | 1.27 | C(24)-C(25) | 1.42 | C(26)-O(31) | 1.29 |
| C(4)-O(5) | 1.39 | C(23)-C(24) | 1.43 | C(26)-O(31) | 1.22 | C(23)-O(36) | 1.39 | C(26)-N(27) | 1.39 |
| C(3)-C(4) | 1.37 | C(22)-C(23) | 1.41 | C(26)-N(27) | 1.27 | C(23)-C(24) | 1.42 | C(25)-H(48) | 1.10 |
| C(2)-C(6) | 1.44 | C(21)-C(22) | 1.40 | C(25)-H(49) | 1.10 | C(22)-C(23) | 1.41 | C(24)-C(26) | 1.46 |
| C(2)-O(5) | 1.40 | C(20)-C(25) | 1.40 | C(24)-C(26) | 1.35 | C(21)-C(22) | 1.40 | C(24)-C(25) | 1.41 |
| C(1)-C(3) | 1.43 | C(20)-C(21) | 1.42 | C(24)-C(25) | 1.34 | C(20)-C(25) | 1.40 | C(23)-O(36) | 1.35 |
| C(1)-C(2) | 1.38 | Cu(19)-O(31) | 1.95 | C(23)-O(36) | 1.38 | C(20)-C(21) | 1.41 | C(23)-C(24) | 1.41 |
|  |  | Cu(19)-N(28) | 2.01 | C(23)-C(24) | 1.35 | Zn(19)-O(31) | 1.94 | C(22)-H(47) | 1.10 |
|  |  | O(17)-H(18) | 1.02 | C(22)-H(48) | 1.10 | Zn(19)-N(28) | 2.03 | C(22)-C(23) | 1.41 |
|  |  | C(15)-O(16) | 1.39 | C(22)-C(23) | 1.34 | O(17)-H(18) | 1.00 | C(21)-H(46) | 1.10 |
|  |  | C(14)-C(15) | 1.38 | C(21)-H(47) | 1.10 | C(15)-O(16) | 1.39 | C(21)-C(22) | 1.38 |
|  |  | C(13)-C(14) | 1.44 | C(21)-C(22) | 1.34 | C(14)-C(15) | 1.38 | C(20)-H(45) | 1.10 |
|  |  | O(12)-Cu(19) | 1.92 | C(20)-H(46) | 1.10 | C(13)-C(14) | 1.43 | C(20)-C(25) | 1.38 |
|  |  | C(11)-O(16) | 1.41 | C(20)-C(25) | 1.34 | O(12)-Zn(19) | 1.97 | C(20)-C(21) | 1.40 |
|  |  | C(11)-C(13) | 1.39 | C(20)-C(21) | 1.34 | C(11)-O(16) | 1.40 | Co(19)-O(35) | 2.11 |
|  |  | C(10)-C(11) | 1.44 | Ni(19)-O(31) | 1.80 | C(11)-C(13) | 1.40 | Co(19)-O(31) | 1.95 |
|  |  | N(9)-Cu(19) | 2.02 | Ni(19)-N(28) | 1.84 | C(10)-C(11) | 1.44 | Co(19)-N(28) | 1.86 |
|  |  | N(9)-C(10) | 1.31 | O(17)-H(18) | 1.00 | N(9)-Zn(19) | 2.03 | O(17)-H(18) | 0.97 |
|  |  | N(8)-N(9) | 1.41 | C(15)-H(45) | 1.10 | N(9)-C(10) | 1.32 | O(16)-Co(19) | 2.11 |
|  |  | C(7)-O(12) | 1.32 | C(15)-O(16) | 1.23 | N(8)-N(9) | 1.40 | C(15)-H(44) | 1.09 |
|  |  | C(7)-N(8) | 1.36 | C(14)-H(44) | 1.10 | C(7)-O(12) | 1.35 | C(15)-O(16) | 1.40 |
|  |  | C(5)-C(7) | 1.47 | C(14)-C(15) | 1.34 | C(7)-N(8) | 1.34 | C(14)-H(43) | 1.09 |
|  |  | C(5)-C(6) | 1.42 | C(13)-H(43) | 1.10 | C(5)-C(7) | 1.47 | C(14)-C(15) | 1.38 |
|  |  | C(4)-O(17) | 1.38 | C(13)-C(14) | 1.34 | C(5)-C(6) | 1.42 | C(13)-H(42) | 1.09 |
|  |  | C(4)-C(5) | 1.43 | O(12)-Ni(19) | 1.80 | C(4)-O(17) | 1.38 | C(13)-C(14) | 1.43 |
|  |  | C(3)-C(4) | 1.41 | C(11)-O(16) | 1.23 | C(4)-C(5) | 1.43 | O(12)-Co(19) | 1.95 |
|  |  | C(2)-C(3) | 1.40 | C(11)-C(13) | 1.34 | C(3)-C(4) | 1.41 | C(11)-O(16) | 1.41 |
|  |  | C(1)-C(6) | 1.40 | C(10)-H(42) | 1.11 | C(2)-C(3) | 1.40 | C(11)-C(13) | 1.39 |
|  |  | C(1)-C(2) | 1.42 | C(10)-C(11) | 1.35 | C(1)-C(6) | 1.40 | C(10)-H(41) | 1.10 |
|  |  |  |  | N(9)-Ni(19) | 1.84 | C(1)-C(2) | 1.42 | C(10)-C(11) | 1.44 |
|  |  |  |  | N(9)-C(10) | 1.27 |  |  | N(9)-Co(19) | 1.86 |
|  |  |  |  | N(8)-H(18) | 1.04 |  |  | N(9)-C(10) | 1.32 |
|  |  |  |  | N(8)-N(9) | 1.26 |  |  | N(8)-H(18) | 1.81 |
|  |  |  |  | C(7)-O(12) | 1.23 |  |  | N(8)-N(9) | 1.35 |
|  |  |  |  | C(7)-N(8) | 1.27 |  |  | C(7)-O(12) | 1.29 |
|  |  |  |  | C(6)-H(41) | 1.10 |  |  | C(7)-N(8) | 1.39 |
|  |  |  |  | C(5)-C(7) | 1.35 |  |  | C(6)-H(40) | 1.10 |
|  |  |  |  | C(5)-C(6) | 1.34 |  |  | C(5)-C(7) | 1.46 |
|  |  |  |  | C(4)-O(17) | 1.38 |  |  | C(5)-C(6) | 1.41 |
|  |  |  |  | C(4)-C(5) | 1.35 |  |  | C(4)-O(17) | 1.35 |
|  |  |  |  | C(3)-H(40) | 1.10 |  |  | C(4)-C(5) | 1.41 |
|  |  |  |  | C(3)-C(4) | 1.34 |  |  | C(3)-H(39) | 1.10 |
|  |  |  |  | C(2)-H(39) | 1.10 |  |  | C(3)-C(4) | 1.41 |
|  |  |  |  | C(2)-C(3) | 1.34 |  |  | C(2)-H(38) | 1.10 |
|  |  |  |  | C(1)-H(38) | 1.10 |  |  | C(2)-C(3) | 1.38 |
|  |  |  |  | C(1)-C(6) | 1.34 |  |  | C(1)-H(37) | 1.10 |
|  |  |  |  | C(1)-C(2) | 1.34 |  |  | C(1)-C(6) | 1.38 |
|  |  |  |  |  |  |  |  | C(1)-C(2) | 1.40 |

**Table S2**. Bond angle data of the ligand and its complexes

| **Ligand** | | Cu | | Ni | | Zn | | Co | |
| --- | --- | --- | --- | --- | --- | --- | --- | --- | --- |
| H(27)-O(17)-C(16) | 109.6 | H(37)-O(36)-C(23) | 108.6 | O(36)-H(37)-N(27) | 165.5 | H(37)-O(36)-C(23) | 111.2 | H(53)-O(36)-C(23) | 108.7 |
| O(17)-C(16)-C(15) | 122.9 | C(34)-O(35)-C(30) | 107.2 | H(37)-O(36)-C(23) | 99.7 | C(34)-O(35)-C(30) | 107.1 | C(34)-O(35)-C(30) | 106.2 |
| O(17)-C(16)-C(14) | 116.8 | O(35)-C(34)-C(33) | 109.9 | C(34)-O(35)-C(30) | 108.9 | O(35)-C(34)-C(33) | 110.5 | C(34)-O(35)-Co(19) | 150.1 |
| C(15)-C(16)-C(14) | 120.2 | C(34)-C(33)-C(32) | 106.9 | H(53)-C(34)-O(35) | 117.4 | C(34)-C(33)-C(32) | 106.4 | C(30)-O(35)-Co(19) | 102.8 |
| C(16)-C(15)-C(13) | 118.0 | C(33)-C(32)-C(30) | 107.2 | H(53)-C(34)-C(33) | 130.9 | C(33)-C(32)-C(30) | 107.5 | H(52)-C(34)-O(35) | 115.0 |
| C(16)-C(15)-C(9) | 118.7 | C(26)-O(31)-Cu(19) | 111.9 | O(35)-C(34)-C(33) | 111.7 | C(26)-O(31)-Zn(19) | 111.0 | H(52)-C(34)-C(33) | 134.9 |
| C(13)-C(15)-C(9) | 123.3 | O(35)-C(30)-C(32) | 108.9 | H(52)-C(33)-C(34) | 128.0 | O(35)-C(30)-C(32) | 108.6 | O(35)-C(34)-C(33) | 110.1 |
| C(16)-C(14)-C(12) | 120.4 | O(35)-C(30)-C(29) | 119.4 | H(52)-C(33)-C(32) | 128.1 | O(35)-C(30)-C(29) | 124.0 | H(51)-C(33)-C(34) | 127.0 |
| C(15)-C(13)-C(11) | 121.5 | C(32)-C(30)-C(29) | 131.7 | C(34)-C(33)-C(32) | 103.9 | C(32)-C(30)-C(29) | 127.4 | H(51)-C(33)-C(32) | 125.6 |
| C(14)-C(12)-C(11) | 120.4 | C(30)-C(29)-N(28) | 124.4 | H(51)-C(32)-C(33) | 127.1 | C(30)-C(29)-N(28) | 131.3 | C(34)-C(33)-C(32) | 107.3 |
| C(13)-C(11)-C(12) | 119.5 | C(29)-N(28)-N(27) | 116.6 | H(51)-C(32)-C(30) | 128.1 | C(29)-N(28)-N(27) | 119.2 | H(50)-C(32)-C(33) | 126.1 |
| C(15)-C(9)-O(10) | 121.2 | C(29)-N(28)-Cu(19) | 131.6 | C(33)-C(32)-C(30) | 104.8 | C(29)-N(28)-Zn(19) | 128.6 | H(50)-C(32)-C(30) | 126.9 |
| C(15)-C(9)-N(8) | 118.2 | N(27)-N(28)-Cu(19) | 111.8 | C(26)-O(31)-Ni(19) | 112.3 | N(27)-N(28)-Zn(19) | 112.2 | C(33)-C(32)-C(30) | 107.0 |
| O(10)-C(9)-N(8) | 120.7 | N(28)-N(27)-C(26) | 111.6 | O(35)-C(30)-C(32) | 110.7 | H(54)-N(27)-N(28) | 124.5 | C(26)-O(31)-Co(19) | 108.5 |
| H(22)-N(8)-C(9) | 119.3 | O(31)-C(26)-N(27) | 121.8 | O(35)-C(30)-C(29) | 124.2 | H(54)-N(27)-C(26) | 124.5 | O(35)-C(30)-C(32) | 109.4 |
| H(22)-N(8)-N(7) | 109.8 | O(31)-C(26)-C(24) | 120.5 | C(32)-C(30)-C(29) | 125.1 | N(28)-N(27)-C(26) | 110.9 | O(35)-C(30)-C(29) | 119.7 |
| C(9)-N(8)-N(7) | 130.9 | N(27)-C(26)-C(24) | 117.7 | H(50)-C(29)-C(30) | 120.0 | O(31)-C(26)-N(27) | 123.9 | C(32)-C(30)-C(29) | 130.9 |
| N(8)-N(7)-C(6) | 121.4 | C(24)-C(25)-C(20) | 120.8 | H(50)-C(29)-N(28) | 110.8 | O(31)-C(26)-C(24) | 116.2 | H(49)-C(29)-C(30) | 119.9 |
| N(7)-C(6)-C(2) | 120.6 | C(26)-C(24)-C(25) | 119.3 | C(30)-C(29)-N(28) | 129.1 | N(27)-C(26)-C(24) | 119.8 | H(49)-C(29)-N(28) | 125.1 |
| C(4)-O(5)-C(2) | 107.0 | C(26)-C(24)-C(23) | 121.4 | C(29)-N(28)-N(27) | 110.8 | C(24)-C(25)-C(20) | 122.1 | C(30)-C(29)-N(28) | 115.0 |
| O(5)-C(4)-C(3) | 110.0 | C(25)-C(24)-C(23) | 119.3 | C(29)-N(28)-Ni(19) | 108.1 | C(26)-C(24)-C(25) | 116.9 | C(29)-N(28)-N(27) | 129.9 |
| C(4)-C(3)-C(1) | 106.8 | O(36)-C(23)-C(24) | 122.3 | N(27)-N(28)-Ni(19) | 117.8 | C(26)-C(24)-C(23) | 125.3 | C(29)-N(28)-Co(19) | 116.5 |
| C(6)-C(2)-O(5) | 120.6 | O(36)-C(23)-C(22) | 118.1 | H(37)-N(27)-N(28) | 123.3 | C(25)-C(24)-C(23) | 117.9 | N(27)-N(28)-Co(19) | 113.6 |
| C(6)-C(2)-C(1) | 130.6 | C(24)-C(23)-C(22) | 119.6 | H(37)-N(27)-C(26) | 104.7 | O(36)-C(23)-C(24) | 119.8 | N(28)-N(27)-C(26) | 112.1 |
| O(5)-C(2)-C(1) | 108.8 | C(23)-C(22)-C(21) | 120.1 | N(28)-N(27)-C(26) | 102.4 | O(36)-C(23)-C(22) | 120.0 | O(31)-C(26)-N(27) | 120.9 |
| C(3)-C(1)-C(2) | 107.4 | C(22)-C(21)-C(20) | 120.8 | O(31)-C(26)-N(27) | 113.9 | C(24)-C(23)-C(22) | 120.1 | O(31)-C(26)-C(24) | 120.6 |
|  |  | C(25)-C(20)-C(21) | 119.4 | O(31)-C(26)-C(24) | 126.9 | C(23)-C(22)-C(21) | 120.8 | N(27)-C(26)-C(24) | 118.5 |
|  |  | O(31)-Cu(19)-N(28) | 81.4 | N(27)-C(26)-C(24) | 119.1 | C(22)-C(21)-C(20) | 119.8 | H(48)-C(25)-C(24) | 119.5 |
|  |  | O(31)-Cu(19)-O(12) | 102.4 | H(49)-C(25)-C(24) | 120.9 | C(25)-C(20)-C(21) | 119.3 | H(48)-C(25)-C(20) | 119.6 |
|  |  | O(31)-Cu(19)-N(9) | 144.1 | H(49)-C(25)-C(20) | 119.9 | O(31)-Zn(19)-N(28) | 81.9 | C(24)-C(25)-C(20) | 120.9 |
|  |  | N(28)-Cu(19)-O(12) | 150.1 | C(24)-C(25)-C(20) | 119.2 | O(31)-Zn(19)-O(12) | 130.8 | C(26)-C(24)-C(25) | 119.2 |
|  |  | N(28)-Cu(19)-N(9) | 112.2 | C(26)-C(24)-C(25) | 125.6 | O(31)-Zn(19)-N(9) | 121.3 | C(26)-C(24)-C(23) | 122.3 |
|  |  | O(12)-Cu(19)-N(9) | 82.2 | C(26)-C(24)-C(23) | 112.8 | N(28)-Zn(19)-O(12) | 120.8 | C(25)-C(24)-C(23) | 118.5 |
|  |  | H(18)-O(17)-C(4) | 108.5 | C(25)-C(24)-C(23) | 121.6 | N(28)-Zn(19)-N(9) | 127.3 | O(36)-C(23)-C(24) | 124.8 |
|  |  | C(15)-O(16)-C(11) | 107.2 | O(36)-C(23)-C(24) | 118.1 | O(12)-Zn(19)-N(9) | 81.3 | O(36)-C(23)-C(22) | 115.1 |
|  |  | O(16)-C(15)-C(14) | 110.0 | O(36)-C(23)-C(22) | 123.6 | H(18)-O(17)-C(4) | 108.7 | C(24)-C(23)-C(22) | 120.1 |
|  |  | C(15)-C(14)-C(13) | 107.0 | C(24)-C(23)-C(22) | 118.2 | C(15)-O(16)-C(11) | 107.0 | H(47)-C(22)-C(23) | 119.6 |
|  |  | C(14)-C(13)-C(11) | 107.4 | H(48)-C(22)-C(23) | 119.4 | O(16)-C(15)-C(14) | 110.4 | H(47)-C(22)-C(21) | 120.6 |
|  |  | Cu(19)-O(12)-C(7) | 111.7 | H(48)-C(22)-C(21) | 120.0 | C(15)-C(14)-C(13) | 106.4 | C(23)-C(22)-C(21) | 119.8 |
|  |  | O(16)-C(11)-C(13) | 108.5 | C(23)-C(22)-C(21) | 120.6 | C(14)-C(13)-C(11) | 107.5 | H(46)-C(21)-C(22) | 119.9 |
|  |  | O(16)-C(11)-C(10) | 113.9 | H(47)-C(21)-C(22) | 119.8 | Zn(19)-O(12)-C(7) | 111.1 | H(46)-C(21)-C(20) | 119.7 |
|  |  | C(13)-C(11)-C(10) | 137.5 | H(47)-C(21)-C(20) | 119.8 | O(16)-C(11)-C(13) | 108.6 | C(22)-C(21)-C(20) | 120.5 |
|  |  | C(11)-C(10)-N(9) | 125.7 | C(22)-C(21)-C(20) | 120.3 | O(16)-C(11)-C(10) | 123.9 | H(45)-C(20)-C(25) | 120.0 |
|  |  | Cu(19)-N(9)-C(10) | 134.2 | H(46)-C(20)-C(25) | 119.9 | C(13)-C(11)-C(10) | 127.5 | H(45)-C(20)-C(21) | 119.8 |
|  |  | Cu(19)-N(9)-N(8) | 110.2 | H(46)-C(20)-C(21) | 120.0 | C(11)-C(10)-N(9) | 131.0 | C(25)-C(20)-C(21) | 120.2 |
|  |  | C(10)-N(9)-N(8) | 115.6 | C(25)-C(20)-C(21) | 120.0 | Zn(19)-N(9)-C(10) | 128.1 | O(35)-Co(19)-O(31) | 169.5 |
|  |  | N(9)-N(8)-C(7) | 112.2 | O(31)-Ni(19)-N(28) | 75.7 | Zn(19)-N(9)-N(8) | 112.7 | O(35)-Co(19)-N(28) | 85.6 |
|  |  | O(12)-C(7)-N(8) | 122.0 | O(31)-Ni(19)-O(12) | 63.3 | C(10)-N(9)-N(8) | 119.1 | O(35)-Co(19)-O(16) | 86.2 |
|  |  | O(12)-C(7)-C(5) | 120.2 | O(31)-Ni(19)-N(9) | 115.6 | N(9)-N(8)-C(7) | 111.8 | O(35)-Co(19)-O(12) | 91.5 |
|  |  | N(8)-C(7)-C(5) | 117.8 | N(28)-Ni(19)-O(12) | 117.0 | O(12)-C(7)-N(8) | 123.1 | O(35)-Co(19)-N(9) | 97.3 |
|  |  | C(5)-C(6)-C(1) | 120.8 | N(28)-Ni(19)-N(9) | 84.2 | O(12)-C(7)-C(5) | 118.0 | O(31)-Co(19)-N(28) | 84.8 |
|  |  | C(7)-C(5)-C(6) | 119.4 | O(12)-Ni(19)-N(9) | 73.9 | N(8)-C(7)-C(5) | 118.9 | O(31)-Co(19)-O(16) | 91.0 |
|  |  | C(7)-C(5)-C(4) | 121.3 | O(17)-H(18)-N(8) | 165.7 | C(5)-C(6)-C(1) | 120.9 | O(31)-Co(19)-O(12) | 93.1 |
|  |  | C(6)-C(5)-C(4) | 119.3 | H(18)-O(17)-C(4) | 99.5 | C(7)-C(5)-C(6) | 120.3 | O(31)-Co(19)-N(9) | 92.5 |
|  |  | O(17)-C(4)-C(5) | 122.2 | C(15)-O(16)-C(11) | 109.0 | C(7)-C(5)-C(4) | 120.7 | N(28)-Co(19)-O(16) | 97.4 |
|  |  | O(17)-C(4)-C(3) | 118.2 | H(45)-C(15)-O(16) | 117.4 | C(6)-C(5)-C(4) | 118.9 | N(28)-Co(19)-O(12) | 92.5 |
|  |  | C(5)-C(4)-C(3) | 119.6 | H(45)-C(15)-C(14) | 130.8 | O(17)-C(4)-C(5) | 122.7 | N(28)-Co(19)-N(9) | 176.0 |
|  |  | C(4)-C(3)-C(2) | 120.1 | O(16)-C(15)-C(14) | 111.8 | O(17)-C(4)-C(3) | 117.4 | O(16)-Co(19)-O(12) | 169.6 |
|  |  | C(3)-C(2)-C(1) | 120.8 | H(44)-C(14)-C(15) | 128.1 | C(5)-C(4)-C(3) | 119.9 | O(16)-Co(19)-N(9) | 85.5 |
|  |  | C(6)-C(1)-C(2) | 119.4 | H(44)-C(14)-C(13) | 128.2 | C(4)-C(3)-C(2) | 120.1 | O(12)-Co(19)-N(9) | 84.8 |
|  |  |  |  | C(15)-C(14)-C(13) | 103.8 | C(3)-C(2)-C(1) | 120.6 | O(17)-H(18)-N(8) | 144.6 |
|  |  |  |  | H(43)-C(13)-C(14) | 126.9 | C(6)-C(1)-C(2) | 119.5 | H(18)-O(17)-C(4) | 108.7 |
|  |  |  |  | H(43)-C(13)-C(11) | 128.1 |  |  | Co(19)-O(16)-C(15) | 150.3 |
|  |  |  |  | C(14)-C(13)-C(11) | 105.0 |  |  | Co(19)-O(16)-C(11) | 103.0 |
|  |  |  |  | Ni(19)-O(12)-C(7) | 114.5 |  |  | C(15)-O(16)-C(11) | 106.2 |
|  |  |  |  | O(16)-C(11)-C(13) | 110.4 |  |  | H(44)-C(15)-O(16) | 115.0 |
|  |  |  |  | O(16)-C(11)-C(10) | 125.2 |  |  | H(44)-C(15)-C(14) | 134.9 |
|  |  |  |  | C(13)-C(11)-C(10) | 124.4 |  |  | O(16)-C(15)-C(14) | 110.1 |
|  |  |  |  | H(42)-C(10)-C(11) | 117.8 |  |  | H(43)-C(14)-C(15) | 127.0 |
|  |  |  |  | H(42)-C(10)-N(9) | 111.1 |  |  | H(43)-C(14)-C(13) | 125.6 |
|  |  |  |  | C(11)-C(10)-N(9) | 131.1 |  |  | C(15)-C(14)-C(13) | 107.3 |
|  |  |  |  | Ni(19)-N(9)-C(10) | 107.9 |  |  | H(42)-C(13)-C(14) | 126.1 |
|  |  |  |  | Ni(19)-N(9)-N(8) | 110.8 |  |  | H(42)-C(13)-C(11) | 126.9 |
|  |  |  |  | C(10)-N(9)-N(8) | 121.8 |  |  | C(14)-C(13)-C(11) | 107.0 |
|  |  |  |  | H(18)-N(8)-N(9) | 125.6 |  |  | Co(19)-O(12)-C(7) | 108.4 |
|  |  |  |  | H(18)-N(8)-C(7) | 104.7 |  |  | O(16)-C(11)-C(13) | 109.4 |
|  |  |  |  | N(9)-N(8)-C(7) | 101.0 |  |  | O(16)-C(11)-C(10) | 119.7 |
|  |  |  |  | O(12)-C(7)-N(8) | 115.5 |  |  | C(13)-C(11)-C(10) | 130.9 |
|  |  |  |  | O(12)-C(7)-C(5) | 125.9 |  |  | H(41)-C(10)-C(11) | 119.9 |
|  |  |  |  | N(8)-C(7)-C(5) | 118.5 |  |  | H(41)-C(10)-N(9) | 125.1 |
|  |  |  |  | H(41)-C(6)-C(5) | 121.0 |  |  | C(11)-C(10)-N(9) | 115.1 |
|  |  |  |  | H(41)-C(6)-C(1) | 119.7 |  |  | Co(19)-N(9)-C(10) | 116.6 |
|  |  |  |  | C(5)-C(6)-C(1) | 119.3 |  |  | Co(19)-N(9)-N(8) | 113.5 |
|  |  |  |  | C(7)-C(5)-C(6) | 125.6 |  |  | C(10)-N(9)-N(8) | 129.9 |
|  |  |  |  | C(7)-C(5)-C(4) | 113.1 |  |  | H(18)-N(8)-N(9) | 146.6 |
|  |  |  |  | C(6)-C(5)-C(4) | 121.3 |  |  | H(18)-N(8)-C(7) | 100.9 |
|  |  |  |  | O(17)-C(4)-C(5) | 118.0 |  |  | N(9)-N(8)-C(7) | 112.1 |
|  |  |  |  | O(17)-C(4)-C(3) | 123.5 |  |  | O(12)-C(7)-N(8) | 120.9 |
|  |  |  |  | C(5)-C(4)-C(3) | 118.4 |  |  | O(12)-C(7)-C(5) | 120.6 |
|  |  |  |  | H(40)-C(3)-C(4) | 119.4 |  |  | N(8)-C(7)-C(5) | 118.5 |
|  |  |  |  | H(40)-C(3)-C(2) | 120.0 |  |  | H(40)-C(6)-C(5) | 119.5 |
|  |  |  |  | C(4)-C(3)-C(2) | 120.6 |  |  | H(40)-C(6)-C(1) | 119.6 |
|  |  |  |  | H(39)-C(2)-C(3) | 119.9 |  |  | C(5)-C(6)-C(1) | 120.9 |
|  |  |  |  | H(39)-C(2)-C(1) | 119.9 |  |  | C(7)-C(5)-C(6) | 119.2 |
|  |  |  |  | C(3)-C(2)-C(1) | 120.3 |  |  | C(7)-C(5)-C(4) | 122.4 |
|  |  |  |  | H(38)-C(1)-C(6) | 120.0 |  |  | C(6)-C(5)-C(4) | 118.5 |
|  |  |  |  | H(38)-C(1)-C(2) | 120.0 |  |  | O(17)-C(4)-C(5) | 124.8 |
|  |  |  |  | C(6)-C(1)-C(2) | 120.1 |  |  | O(17)-C(4)-C(3) | 115.1 |
|  |  |  |  |  |  |  |  | C(5)-C(4)-C(3) | 120.1 |
|  |  |  |  |  |  |  |  | H(39)-C(3)-C(4) | 119.6 |
|  |  |  |  |  |  |  |  | H(39)-C(3)-C(2) | 120.6 |
|  |  |  |  |  |  |  |  | C(4)-C(3)-C(2) | 119.8 |
|  |  |  |  |  |  |  |  | H(38)-C(2)-C(3) | 119.9 |
|  |  |  |  |  |  |  |  | H(38)-C(2)-C(1) | 119.7 |
|  |  |  |  |  |  |  |  | C(3)-C(2)-C(1) | 120.5 |
|  |  |  |  |  |  |  |  | H(37)-C(1)-C(6) | 120.0 |
|  |  |  |  |  |  |  |  | H(37)-C(1)-C(2) | 119.8 |
|  |  |  |  |  |  |  |  | C(6)-C(1)-C(2) | 120.2 |

**Table S3**. Dihedral angle data of the ligand and its complexes

|  | **H_2_L** |  | **Cu** |  | **Ni** |
| --- | --- | --- | --- | --- | --- |
| C3(Ph)-C2(Ph)-OH(Ph)-H(27) | 176.4 | C(32)-C(33)-O(34)-C(29) | 0.7 | C6(Ph1)-C(1)-C(2) | 120.2 |
| C1(Ph)-C2(Ph)-OH(Ph)-H(27) | -3.5 | C(31)-C(32)-C(33)-O(34) | -0.1 | C(32)-C(33)-O(34)-Ni(18) | -144.3 |
| C1(Ket)-C1(Ph)-C2(Ph)-C3(Ph) | 178.0 | C(29)-C(31)-C(32)-C(33) | -0.6 | C(32)-C(33)-O(34)-C(29) | 1.3 |
| C1(Ket)-C1(Ph)-C2(Ph)-OH(Ph) | -2.1 | C(28)-C(29)-O(34)-C(33) | -179.5 | H(52)-C(33)-O(34)-Ni(18) | 34.9 |
| C6(Ph)-C1(Ph)-C2(Ph)-C3(Ph) | -2.0 | C(31)-C(29)-O(34)-C(33) | -1.0 | H(52)-C(33)-O(34)-C(29) | -179.6 |
| C6(Ph)-C1(Ph)-C2(Ph)-OH(Ph) | 177.9 | C(28)-C(29)-C(31)-C(32) | 179.2 | C(31)-C(32)-C(33)-O(34) | -0.5 |
| C4(Ph)-C3(Ph)-C2(Ph)-C1(Ph) | 1.0 | O(34)-C(29)-C(31)-C(32) | 1.0 | C(31)-C(32)-C(33)-H(52) | -179.5 |
| C4(Ph)-C3(Ph)-C2(Ph)-OH(Ph) | -178.9 | N(27)-C(28)-C(29)-C(31) | 168.6 | H(51)-C(32)-C(33)-O(34) | -179.6 |
| C5(Ph)-C6(Ph)-C1(Ph)-C1(Ket) | -178.4 | N(27)-C(28)-C(29)-O(34) | -13.3 | H(51)-C(32)-C(33)-H(52) | 1.4 |
| C5(Ph)-C6(Ph)-C1(Ph)-C2(Ph) | 1.6 | Cu(18)-N(27)-C(28)-C(29) | -10.1 | C(29)-C(31)-C(32)-C(33) | -0.5 |
| C5(Ph)-C4(Ph)-C3(Ph)-C2(Ph) | 0.4 | N(26)-N(27)-C(28)-C(29) | 174.2 | C(29)-C(31)-C(32)-H(51) | 178.6 |
| C4(Ph)-C5(Ph)-C6(Ph)-C1(Ph) | -0.2 | C(25)-N(26)-N(27)-Cu(18) | 8.0 | H(50)-C(31)-C(32)-C(33) | -179.7 |
| C6(Ph)-C5(Ph)-C4(Ph)-C3(Ph) | -0.8 | C(25)-N(26)-N(27)-C(28) | -175.5 | H(50)-C(31)-C(32)-H(51) | -0.5 |
| N1-C1(Ket)-C1(Ph)-C6(Ph) | 10.7 | C(23)-C(25)-O(30)-Cu(18) | 174.1 | C(28)-C(29)-O(34)-Ni(18) | -22.4 |
| N1-C1(Ket)-C1(Ph)-C2(Ph) | -169.3 | N(26)-C(25)-O(30)-Cu(18) | -6.2 | C(28)-C(29)-O(34)-C(33) | 174.3 |
| O(Ket)-C1(Ket)-C1(Ph)-C6(Ph) | -170.3 | C(23)-C(25)-N(26)-N(27) | 178.5 | C(31)-C(29)-O(34)-Ni(18) | 161.8 |
| O(Ket)-C1(Ket)-C1(Ph)-C2(Ph) | 9.8 | O(30)-C(25)-N(26)-N(27) | -1.2 | C(31)-C(29)-O(34)-C(33) | -1.6 |
| N2-N1-C1(Ket)-O(Ket) | 5.2 | C(22)-C(23)-C(25)-N(26) | -178.7 | C(28)-C(29)-C(31)-C(32) | -173.9 |
| N2-N1-C1(Ket)-C1(Ph) | -175.7 | C(22)-C(23)-C(25)-O(30) | 1.1 | C(28)-C(29)-C(31)-H(50) | 5.3 |
| H(22)-N1-C1(Ket)-O(Ket) | -175.4 | C(24)-C(23)-C(25)-N(26) | 1.3 | O(34)-C(29)-C(31)-C(32) | 1.4 |
| H(22)-N1-C1(Ket)-C1(Ph) | 3.7 | C(24)-C(23)-C(25)-O(30) | -178.9 | O(34)-C(29)-C(31)-H(50) | -179.5 |
| C1(Meth)-N2-N1-C1(Ket) | -2.3 | C(22)-C(23)-C(24)-C(19) | 0.0 | N(27)-C(28)-C(29)-C(31) | -177.6 |
| C1(Meth)-N2-N1-H(22) | 178.3 | C(25)-C(23)-C(24)-C(19) | -180.0 | N(27)-C(28)-C(29)-O(34) | 7.6 |
| C2(Fur)-C1(Meth)-N2-N1 | 179.5 | C(21)-C(22)-O(35)-H(53) | 179.0 | H(49)-C(28)-C(29)-C(31) | 7.0 |
| C4(Fur)-C5(Fur)-O(Fur)-C2(Fur) | 0.0 | C(23)-C(22)-O(35)-H(53) | -1.0 | H(49)-C(28)-C(29)-O(34) | -167.8 |
| C3(Fur)-C4(Fur)-C5(Fur)-O(Fur) | 0.0 | C(21)-C(22)-C(23)-C(24) | -0.1 | Ni(18)-N(27)-C(28)-C(29) | 13.6 |
| C3(Fur)-C2(Fur)-C1(Meth)-N2 | 179.9 | C(21)-C(22)-C(23)-C(25) | 179.9 | Ni(18)-N(27)-C(28)-H(49) | -170.8 |
| O(Fur)-C2(Fur)-C1(Meth)-N2 | -0.1 | O(35)-C(22)-C(23)-C(24) | -180.0 | N(26)-N(27)-C(28)-C(29) | -100.6 |
| C3(Fur)-C2(Fur)-O(Fur)-C5(Fur) | 0.0 | O(35)-C(22)-C(23)-C(25) | 0.0 | N(26)-N(27)-C(28)-H(49) | 74.9 |
| C1(Meth)-C2(Fur)-O(Fur)-C5(Fur) | 180.0 | C(20)-C(21)-C(22)-C(23) | 0.1 | C(25)-N(26)-N(27)-Ni(18) | -9.8 |
| C2(Fur)-C3(Fur)-C4(Fur)-C5(Fur) | 0.0 | C(20)-C(21)-C(22)-O(35) | 180.0 | C(25)-N(26)-N(27)-C(28) | 103.5 |
| C4(Fur)-C3(Fur)-C2(Fur)-O(Fur) | 0.0 | C(19)-C(20)-C(21)-C(22) | 0.0 | C(23)-C(25)-O(30)-Ni(18) | -164.5 |
| C4(Fur)-C3(Fur)-C2(Fur)-C1(Meth) | -179.9 | C(20)-C(19)-C(24)-C(23) | 0.0 | N(26)-C(25)-O(30)-Ni(18) | 17.5 |
|  |  | C(24)-C(19)-C(20)-C(21) | 0.0 | C(23)-C(25)-N(26)-N(27) | 176.9 |
|  |  | N(9)-Cu(18)-O(30)-C(25) | 177.2 | O(30)-C(25)-N(26)-N(27) | -5.3 |
|  |  | O(12)-Cu(18)-O(30)-C(25) | -91.6 | C(22)-C(23)-C(25)-N(26) | 160.5 |
|  |  | N(27)-Cu(18)-O(30)-C(25) | 7.9 | C(22)-C(23)-C(25)-O(30) | -17.4 |
|  |  | N(9)-Cu(18)-N(27)-N(26) | -121.2 | C(24)-C(23)-C(25)-N(26) | -19.1 |
|  |  | N(9)-Cu(18)-N(27)-C(28) | 63.0 | C(24)-C(23)-C(25)-O(30) | 163.1 |
|  |  | O(12)-Cu(18)-N(27)-N(26) | 128.7 | C(22)-C(23)-C(24)-C(19) | 0.1 |
|  |  | O(12)-Cu(18)-N(27)-C(28) | -47.1 | C(22)-C(23)-C(24)-H(48) | -179.7 |
|  |  | O(30)-Cu(18)-N(27)-N(26) | -9.0 | C(25)-C(23)-C(24)-C(19) | 179.7 |
|  |  | O(30)-Cu(18)-N(27)-C(28) | 175.2 | C(25)-C(23)-C(24)-H(48) | -0.1 |
|  |  | C(14)-C(15)-O(16)-C(11) | 0.7 | C(21)-C(22)-O(35)-H(53) | -177.2 |
|  |  | C(13)-C(14)-C(15)-O(16) | -0.1 | C(23)-C(22)-O(35)-H(53) | 3.2 |
|  |  | C(11)-C(13)-C(14)-C(15) | -0.6 | C(21)-C(22)-C(23)-C(24) | 0.0 |
|  |  | C(7)-O(12)-Cu(18)-N(9) | 7.9 | C(21)-C(22)-C(23)-C(25) | -179.5 |
|  |  | C(7)-O(12)-Cu(18)-N(27) | 177.2 | O(35)-C(22)-C(23)-C(24) | 179.6 |
|  |  | C(7)-O(12)-Cu(18)-O(30) | -91.6 | O(35)-C(22)-C(23)-C(25) | 0.0 |
|  |  | C(10)-C(11)-O(16)-C(15) | -179.5 | C(20)-C(21)-C(22)-C(23) | -0.1 |
|  |  | C(13)-C(11)-O(16)-C(15) | -1.0 | C(20)-C(21)-C(22)-O(35) | -179.7 |
|  |  | C(10)-C(11)-C(13)-C(14) | 179.2 | H(47)-C(21)-C(22)-C(23) | 179.9 |
|  |  | O(16)-C(11)-C(13)-C(14) | 1.0 | H(47)-C(21)-C(22)-O(35) | 0.4 |
|  |  | N(9)-C(10)-C(11)-C(13) | 168.6 | C(19)-C(20)-C(21)-C(22) | 0.1 |
|  |  | N(9)-C(10)-C(11)-O(16) | -13.3 | C(19)-C(20)-C(21)-H(47) | -180.0 |
|  |  | N(8)-N(9)-Cu(18)-O(12) | -9.0 | H(46)-C(20)-C(21)-C(22) | -179.9 |
|  |  | N(8)-N(9)-Cu(18)-N(27) | -121.2 | H(46)-C(20)-C(21)-H(47) | 0.1 |
|  |  | N(8)-N(9)-Cu(18)-O(30) | 128.7 | C(20)-C(19)-C(24)-C(23) | -0.1 |
|  |  | C(10)-N(9)-Cu(18)-O(12) | 175.2 | C(20)-C(19)-C(24)-H(48) | 179.6 |
|  |  | C(10)-N(9)-Cu(18)-N(27) | 63.0 | H(45)-C(19)-C(24)-C(23) | 179.9 |
|  |  | C(10)-N(9)-Cu(18)-O(30) | -47.1 | H(45)-C(19)-C(24)-H(48) | -0.3 |
|  |  | N(8)-N(9)-C(10)-C(11) | 174.2 | C(24)-C(19)-C(20)-C(21) | 0.0 |
|  |  | Cu(18)-N(9)-C(10)-C(11) | -10.1 | C(24)-C(19)-C(20)-H(46) | -180.0 |
|  |  | C(7)-N(8)-N(9)-C(10) | -175.5 | H(45)-C(19)-C(20)-C(21) | 179.9 |
|  |  | C(7)-N(8)-N(9)-Cu(18) | 8.0 | H(45)-C(19)-C(20)-H(46) | -0.1 |
|  |  | C1(Ph1)-C(7)-O(12)-Cu(18) | 174.1 | N(9)-Ni(18)-O(34)-C(29) | -153.7 |
|  |  | N(8)-C(7)-O(12)-Cu(18) | -6.2 | N(9)-Ni(18)-O(34)-C(33) | -7.2 |
|  |  | C1(Ph1)-C(7)-N(8)-N(9) | 178.6 | O(12)-Ni(18)-O(34)-C(29) | 132.2 |
|  |  | O(12)-C(7)-N(8)-N(9) | -1.2 | O(12)-Ni(18)-O(34)-C(33) | -81.3 |
|  |  | C2(Ph1)-C1(Ph1)-C(7)-N(8) | -178.7 | O(16)-Ni(18)-O(34)-C(29) | -69.1 |
|  |  | C2(Ph1)-C1(Ph1)-C(7)-O(12) | 1.1 | O(16)-Ni(18)-O(34)-C(33) | 77.4 |
|  |  | C6(Ph1)-C1(Ph1)-C(7)-N(8) | 1.3 | N(27)-Ni(18)-O(34)-C(29) | 24.3 |
|  |  | C6(Ph1)-C1(Ph1)-C(7)-O(12) | -178.9 | N(27)-Ni(18)-O(34)-C(33) | 170.8 |
|  |  | C2(Ph1)-C1(Ph1)-C6(Ph1)-C(1) | 0.0 | O(30)-Ni(18)-O(34)-C(29) | 113.7 |
|  |  | C(7)-C1(Ph1)-C6(Ph1)-C(1) | -180.0 | O(30)-Ni(18)-O(34)-C(33) | -99.8 |
|  |  | C3(Ph1)-C2(Ph1)-O(17)-H(44) | 179.0 | N(9)-Ni(18)-O(30)-C(25) | 155.2 |
|  |  | C1(Ph1)-C2(Ph1)-O(17)-H(44) | -1.0 | O(12)-Ni(18)-O(30)-C(25) | 73.2 |
|  |  | C3(Ph1)-C2(Ph1)-C1(Ph1)-C6(Ph1) | -0.1 | O(16)-Ni(18)-O(30)-C(25) | 165.5 |
|  |  | C3(Ph1)-C2(Ph1)-C1(Ph1)-C(7) | 179.9 | N(27)-Ni(18)-O(30)-C(25) | -18.2 |
|  |  | O(17)-C2(Ph1)-C1(Ph1)-C6(Ph1) | -180.0 | O(34)-Ni(18)-O(30)-C(25) | -109.8 |
|  |  | O(17)-C2(Ph1)-C1(Ph1)-C(7) | 0.0 | N(9)-Ni(18)-N(27)-N(26) | -97.5 |
|  |  | C(2)-C3(Ph1)-C2(Ph1)-C1(Ph1) | 0.1 | N(9)-Ni(18)-N(27)-C(28) | 142.9 |
|  |  | C(2)-C3(Ph1)-C2(Ph1)-O(17) | 180.0 | O(12)-Ni(18)-N(27)-N(26) | -72.6 |
|  |  | C(1)-C(2)-C3(Ph1)-C2(Ph1) | 0.0 | O(12)-Ni(18)-N(27)-C(28) | 167.7 |
|  |  | C(2)-C(1)-C6(Ph1)-C1(Ph1) | 0.0 | O(16)-Ni(18)-N(27)-N(26) | -164.3 |
|  |  | C6(Ph1)-C(1)-C(2)-C3(Ph1) | 0.0 | O(16)-Ni(18)-N(27)-C(28) | 76.0 |
|  |  |  |  | O(30)-Ni(18)-N(27)-N(26) | 15.8 |
|  |  |  |  | O(30)-Ni(18)-N(27)-C(28) | -103.8 |
|  |  |  |  | O(34)-Ni(18)-N(27)-N(26) | 98.3 |
|  |  |  |  | O(34)-Ni(18)-N(27)-C(28) | -21.3 |
|  |  |  |  | C(11)-O(16)-Ni(18)-N(9) | 40.2 |
|  |  |  |  | C(11)-O(16)-Ni(18)-O(12) | 122.1 |
|  |  |  |  | C(11)-O(16)-Ni(18)-N(27) | -146.4 |
|  |  |  |  | C(11)-O(16)-Ni(18)-O(30) | 29.9 |
|  |  |  |  | C(11)-O(16)-Ni(18)-O(34) | -54.4 |
|  |  |  |  | C(15)-O(16)-Ni(18)-N(9) | 160.5 |
|  |  |  |  | C(15)-O(16)-Ni(18)-O(12) | -117.6 |
|  |  |  |  | C(15)-O(16)-Ni(18)-N(27) | -26.1 |
|  |  |  |  | C(15)-O(16)-Ni(18)-O(30) | 150.2 |
|  |  |  |  | C(15)-O(16)-Ni(18)-O(34) | 65.9 |
|  |  |  |  | C(14)-C(15)-O(16)-C(11) | -1.8 |
|  |  |  |  | C(14)-C(15)-O(16)-Ni(18) | -119.3 |
|  |  |  |  | H(43)-C(15)-O(16)-C(11) | -180.0 |
|  |  |  |  | H(43)-C(15)-O(16)-Ni(18) | 62.6 |
|  |  |  |  | C(13)-C(14)-C(15)-O(16) | 1.3 |
|  |  |  |  | C(13)-C(14)-C(15)-H(43) | 179.0 |
|  |  |  |  | H(42)-C(14)-C(15)-O(16) | -178.0 |
|  |  |  |  | H(42)-C(14)-C(15)-H(43) | -0.3 |
|  |  |  |  | C(11)-C(13)-C(14)-C(15) | -0.2 |
|  |  |  |  | C(11)-C(13)-C(14)-H(42) | 179.1 |
|  |  |  |  | H(41)-C(13)-C(14)-C(15) | 179.6 |
|  |  |  |  | H(41)-C(13)-C(14)-H(42) | -1.2 |
|  |  |  |  | C(7)-O(12)-Ni(18)-N(9) | -32.1 |
|  |  |  |  | C(7)-O(12)-Ni(18)-O(16) | -115.8 |
|  |  |  |  | C(7)-O(12)-Ni(18)-N(27) | 150.9 |
|  |  |  |  | C(7)-O(12)-Ni(18)-O(30) | 61.4 |
|  |  |  |  | C(7)-O(12)-Ni(18)-O(34) | 43.0 |
|  |  |  |  | C(10)-C(11)-O(16)-C(15) | 173.4 |
|  |  |  |  | C(10)-C(11)-O(16)-Ni(18) | -47.1 |
|  |  |  |  | C(13)-C(11)-O(16)-C(15) | 1.7 |
|  |  |  |  | C(13)-C(11)-O(16)-Ni(18) | 141.1 |
|  |  |  |  | C(10)-C(11)-C(13)-C(14) | -169.8 |
|  |  |  |  | C(10)-C(11)-C(13)-H(41) | 10.4 |
|  |  |  |  | O(16)-C(11)-C(13)-C(14) | -0.9 |
|  |  |  |  | O(16)-C(11)-C(13)-H(41) | 179.3 |
|  |  |  |  | N(9)-C(10)-C(11)-C(13) | -163.5 |
|  |  |  |  | N(9)-C(10)-C(11)-O(16) | 27.9 |
|  |  |  |  | H(40)-C(10)-C(11)-C(13) | 25.4 |
|  |  |  |  | H(40)-C(10)-C(11)-O(16) | -143.2 |
|  |  |  |  | N(8)-N(9)-Ni(18)-O(12) | 29.1 |
|  |  |  |  | N(8)-N(9)-Ni(18)-O(16) | 121.6 |
|  |  |  |  | N(8)-N(9)-Ni(18)-N(27) | 54.2 |
|  |  |  |  | N(8)-N(9)-Ni(18)-O(30) | -58.9 |
|  |  |  |  | N(8)-N(9)-Ni(18)-O(34) | -141.6 |
|  |  |  |  | C(10)-N(9)-Ni(18)-O(12) | -122.6 |
|  |  |  |  | C(10)-N(9)-Ni(18)-O(16) | -30.1 |
|  |  |  |  | C(10)-N(9)-Ni(18)-N(27) | -97.5 |
|  |  |  |  | C(10)-N(9)-Ni(18)-O(30) | 149.4 |
|  |  |  |  | C(10)-N(9)-Ni(18)-O(34) | 66.7 |
|  |  |  |  | N(8)-N(9)-C(10)-C(11) | -136.6 |
|  |  |  |  | N(8)-N(9)-C(10)-H(40) | 33.9 |
|  |  |  |  | Ni(18)-N(9)-C(10)-C(11) | 8.8 |
|  |  |  |  | Ni(18)-N(9)-C(10)-H(40) | 179.4 |
|  |  |  |  | C(7)-N(8)-N(9)-C(10) | 126.7 |
|  |  |  |  | C(7)-N(8)-N(9)-Ni(18) | -19.0 |
|  |  |  |  | C1(Ph1)-C(7)-O(12)-Ni(18) | -153.1 |
|  |  |  |  | N(8)-C(7)-O(12)-Ni(18) | 31.6 |
|  |  |  |  | C1(Ph1)-C(7)-N(8)-N(9) | 174.8 |
|  |  |  |  | O(12)-C(7)-N(8)-N(9) | -9.8 |
|  |  |  |  | C2(Ph1)-C1(Ph1)-C(7)-N(8) | 2.8 |
|  |  |  |  | C2(Ph1)-C1(Ph1)-C(7)-O(12) | -172.5 |
|  |  |  |  | C6(Ph1)-C1(Ph1)-C(7)-N(8) | -176.6 |
|  |  |  |  | C6(Ph1)-C1(Ph1)-C(7)-O(12) | 8.1 |
|  |  |  |  | C2(Ph1)-C1(Ph1)-C6(Ph1)-C(1) | 0.3 |
|  |  |  |  | C2(Ph1)-C1(Ph1)-C6(Ph1)-H(39) | -179.3 |
|  |  |  |  | C(7)-C1(Ph1)-C6(Ph1)-C(1) | 179.7 |
|  |  |  |  | C(7)-C1(Ph1)-C6(Ph1)-H(39) | 0.1 |
|  |  |  |  | C3(Ph1)-C2(Ph1)-O(17)-H(44) | 177.7 |
|  |  |  |  | C1(Ph1)-C2(Ph1)-O(17)-H(44) | -2.7 |
|  |  |  |  | C3(Ph1)-C2(Ph1)-C1(Ph1)-C6(Ph1) | -0.3 |
|  |  |  |  | C3(Ph1)-C2(Ph1)-C1(Ph1)-C(7) | -179.7 |
|  |  |  |  | O(17)-C2(Ph1)-C1(Ph1)-C6(Ph1) | -179.9 |
|  |  |  |  | O(17)-C2(Ph1)-C1(Ph1)-C(7) | 0.7 |
|  |  |  |  | C(2)-C3(Ph1)-C2(Ph1)-C1(Ph1) | 0.2 |
|  |  |  |  | C(2)-C3(Ph1)-C2(Ph1)-O(17) | 179.8 |
|  |  |  |  | H(38)-C3(Ph1)-C2(Ph1)-C1(Ph1) | -180.0 |
|  |  |  |  | H(38)-C3(Ph1)-C2(Ph1)-O(17) | -0.4 |
|  |  |  |  | C(1)-C(2)-C3(Ph1)-C2(Ph1) | 0.0 |
|  |  |  |  | C(1)-C(2)-C3(Ph1)-H(38) | -179.8 |
|  |  |  |  | H(37)-C(2)-C3(Ph1)-C2(Ph1) | 179.9 |
|  |  |  |  | H(37)-C(2)-C3(Ph1)-H(38) | 0.1 |
|  |  |  |  | C(2)-C(1)-C6(Ph1)-C1(Ph1) | -0.1 |
|  |  |  |  | C(2)-C(1)-C6(Ph1)-H(39) | 179.5 |
|  |  |  |  | H(36)-C(1)-C6(Ph1)-C1(Ph1) | -179.8 |
|  |  |  |  | H(36)-C(1)-C6(Ph1)-H(39) | -0.3 |
|  |  |  |  | C6(Ph1)-C(1)-C(2)-C3(Ph1) | -0.1 |
|  |  |  |  | C6(Ph1)-C(1)-C(2)-H(37) | -179.9 |
|  |  |  |  | H(36)-C(1)-C(2)-C3(Ph1) | 179.7 |
|  |  |  |  | H(36)-C(1)-C(2)-H(37) | -0.2 |
|  | Zn |  | Co |  |  |
| C(32)-C(33)-O(34)-Zn(18) | 127.6 | C6(Ph1)-C(1)-C(2) | 120.2 |  |  |
| C(32)-C(33)-O(34)-C(29) | 0.7 | C(33)-C(34)-O(35)-Co(19) | 165.4 |  |  |
| H(52)-C(33)-O(34)-Zn(18) | -52.5 | C(33)-C(34)-O(35)-C(30) | 0.2 |  |  |
| H(52)-C(33)-O(34)-C(29) | -179.4 | H(52)-C(34)-O(35)-Co(19) | -14.4 |  |  |
| C(31)-C(32)-C(33)-O(34) | -0.3 | H(52)-C(34)-O(35)-C(30) | -179.6 |  |  |
| C(31)-C(32)-C(33)-H(52) | 179.8 | C(32)-C(33)-C(34)-O(35) | -0.1 |  |  |
| H(51)-C(32)-C(33)-O(34) | 179.9 | C(32)-C(33)-C(34)-H(52) | 179.7 |  |  |
| H(51)-C(32)-C(33)-H(52) | 0.0 | H(51)-C(33)-C(34)-O(35) | -179.9 |  |  |
| C(29)-C(31)-C(32)-C(33) | -0.2 | H(51)-C(33)-C(34)-H(52) | -0.1 |  |  |
| C(29)-C(31)-C(32)-H(51) | 179.6 | C(30)-C(32)-C(33)-C(34) | -0.1 |  |  |
| H(50)-C(31)-C(32)-C(33) | -179.6 | C(30)-C(32)-C(33)-H(51) | 179.7 |  |  |
| H(50)-C(31)-C(32)-H(51) | 0.2 | H(50)-C(32)-C(33)-C(34) | -179.7 |  |  |
| C(28)-C(29)-O(34)-Zn(18) | 24.9 | H(50)-C(32)-C(33)-H(51) | 0.1 |  |  |
| C(28)-C(29)-O(34)-C(33) | 179.1 | C(29)-C(30)-O(35)-Co(19) | 6.6 |  |  |
| C(31)-C(29)-O(34)-Zn(18) | -155.0 | C(29)-C(30)-O(35)-C(34) | 179.1 |  |  |
| C(31)-C(29)-O(34)-C(33) | -0.8 | C(32)-C(30)-O(35)-Co(19) | -172.8 |  |  |
| C(28)-C(29)-C(31)-C(32) | -179.2 | C(32)-C(30)-O(35)-C(34) | -0.3 |  |  |
| C(28)-C(29)-C(31)-H(50) | 0.2 | C(29)-C(30)-C(32)-C(33) | -179.0 |  |  |
| O(34)-C(29)-C(31)-C(32) | 0.6 | C(29)-C(30)-C(32)-H(50) | 0.6 |  |  |
| O(34)-C(29)-C(31)-H(50) | -180.0 | O(35)-C(30)-C(32)-C(33) | 0.2 |  |  |
| N(27)-C(28)-C(29)-C(31) | 155.1 | O(35)-C(30)-C(32)-H(50) | 179.8 |  |  |
| N(27)-C(28)-C(29)-O(34) | -24.7 | N(28)-C(29)-C(30)-C(32) | 173.6 |  |  |
| H(49)-C(28)-C(29)-C(31) | -23.7 | N(28)-C(29)-C(30)-O(35) | -5.6 |  |  |
| H(49)-C(28)-C(29)-O(34) | 156.5 | H(49)-C(29)-C(30)-C(32) | -5.7 |  |  |
| Zn(18)-N(27)-C(28)-C(29) | -0.1 | H(49)-C(29)-C(30)-O(35) | 175.1 |  |  |
| Zn(18)-N(27)-C(28)-H(49) | 178.6 | Co(19)-N(28)-C(29)-C(30) | 0.8 |  |  |
| N(26)-N(27)-C(28)-C(29) | 179.9 | Co(19)-N(28)-C(29)-H(49) | -180.0 |  |  |
| N(26)-N(27)-C(28)-H(49) | -1.4 | N(27)-N(28)-C(29)-C(30) | -179.6 |  |  |
| C(25)-N(26)-N(27)-Zn(18) | -1.7 | N(27)-N(28)-C(29)-H(49) | -0.4 |  |  |
| C(25)-N(26)-N(27)-C(28) | 178.3 | C(26)-N(27)-N(28)-Co(19) | -1.1 |  |  |
| C(23)-C(25)-O(30)-Zn(18) | -179.0 | C(26)-N(27)-N(28)-C(29) | 179.2 |  |  |
| N(26)-C(25)-O(30)-Zn(18) | 0.7 | C(24)-C(26)-O(31)-Co(19) | 174.8 |  |  |
| C(23)-C(25)-N(26)-N(27) | -179.6 | N(27)-C(26)-O(31)-Co(19) | -6.0 |  |  |
| O(30)-C(25)-N(26)-N(27) | 0.7 | C(24)-C(26)-N(27)-N(28) | -175.7 |  |  |
| C(22)-C(23)-C(25)-N(26) | -179.8 | O(31)-C(26)-N(27)-N(28) | 5.0 |  |  |
| C(22)-C(23)-C(25)-O(30) | -0.1 | C(23)-C(24)-C(26)-N(27) | 0.2 |  |  |
| C(24)-C(23)-C(25)-N(26) | 0.4 | C(23)-C(24)-C(26)-O(31) | 179.5 |  |  |
| C(24)-C(23)-C(25)-O(30) | -179.9 | C(25)-C(24)-C(26)-N(27) | 179.8 |  |  |
| C(22)-C(23)-C(24)-C(19) | 0.3 | C(25)-C(24)-C(26)-O(31) | -1.0 |  |  |
| C(22)-C(23)-C(24)-H(48) | -179.6 | C(23)-C(24)-C(25)-C(20) | -0.1 |  |  |
| C(25)-C(23)-C(24)-C(19) | -179.9 | C(23)-C(24)-C(25)-H(48) | 179.8 |  |  |
| C(25)-C(23)-C(24)-H(48) | 0.2 | C(26)-C(24)-C(25)-C(20) | -179.6 |  |  |
| C(21)-C(22)-O(35)-H(53) | 179.0 | C(26)-C(24)-C(25)-H(48) | 0.2 |  |  |
| C(23)-C(22)-O(35)-H(53) | -1.1 | C(22)-C(23)-O(36)-H(53) | -178.9 |  |  |
| C(21)-C(22)-C(23)-C(24) | -0.4 | C(24)-C(23)-O(36)-H(53) | 1.3 |  |  |
| C(21)-C(22)-C(23)-C(25) | 179.8 | C(22)-C(23)-C(24)-C(25) | 0.1 |  |  |
| O(35)-C(22)-C(23)-C(24) | 179.8 | C(22)-C(23)-C(24)-C(26) | 179.6 |  |  |
| O(35)-C(22)-C(23)-C(25) | 0.0 | O(36)-C(23)-C(24)-C(25) | 179.9 |  |  |
| C(20)-C(21)-C(22)-C(23) | 0.2 | O(36)-C(23)-C(24)-C(26) | -0.5 |  |  |
| C(20)-C(21)-C(22)-O(35) | -179.9 | C(21)-C(22)-C(23)-C(24) | -0.1 |  |  |
| H(47)-C(21)-C(22)-C(23) | -179.9 | C(21)-C(22)-C(23)-O(36) | -179.9 |  |  |
| H(47)-C(21)-C(22)-O(35) | -0.1 | H(47)-C(22)-C(23)-C(24) | -180.0 |  |  |
| C(19)-C(20)-C(21)-C(22) | 0.0 | H(47)-C(22)-C(23)-O(36) | 0.2 |  |  |
| C(19)-C(20)-C(21)-H(47) | -179.9 | C(20)-C(21)-C(22)-C(23) | 0.0 |  |  |
| H(46)-C(20)-C(21)-C(22) | 179.9 | C(20)-C(21)-C(22)-H(47) | 179.9 |  |  |
| H(46)-C(20)-C(21)-H(47) | 0.1 | H(46)-C(21)-C(22)-C(23) | -179.9 |  |  |
| C(20)-C(19)-C(24)-C(23) | -0.1 | H(46)-C(21)-C(22)-H(47) | 0.0 |  |  |
| C(20)-C(19)-C(24)-H(48) | 179.8 | C(21)-C(20)-C(25)-C(24) | 0.0 |  |  |
| H(45)-C(19)-C(24)-C(23) | -180.0 | C(21)-C(20)-C(25)-H(48) | -179.8 |  |  |
| H(45)-C(19)-C(24)-H(48) | 0.0 | H(45)-C(20)-C(25)-C(24) | 179.9 |  |  |
| C(24)-C(19)-C(20)-C(21) | 0.0 | H(45)-C(20)-C(25)-H(48) | 0.1 |  |  |
| C(24)-C(19)-C(20)-H(46) | -180.0 | C(25)-C(20)-C(21)-C(22) | 0.0 |  |  |
| H(45)-C(19)-C(20)-C(21) | 179.8 | C(25)-C(20)-C(21)-H(46) | 179.9 |  |  |
| H(45)-C(19)-C(20)-H(46) | -0.1 | H(45)-C(20)-C(21)-C(22) | -179.9 |  |  |
| N(9)-Zn(18)-O(34)-C(29) | -171.8 | H(45)-C(20)-C(21)-H(46) | 0.0 |  |  |
| N(9)-Zn(18)-O(34)-C(33) | 58.8 | N(9)-Co(19)-O(35)-C(30) | 172.6 |  |  |
| O(12)-Zn(18)-O(34)-C(29) | 104.6 | N(9)-Co(19)-O(35)-C(34) | 7.2 |  |  |
| O(12)-Zn(18)-O(34)-C(33) | -24.8 | O(12)-Co(19)-O(35)-C(30) | 87.7 |  |  |
| O(16)-Zn(18)-O(34)-C(29) | -103.5 | O(12)-Co(19)-O(35)-C(34) | -77.7 |  |  |
| O(16)-Zn(18)-O(34)-C(33) | 127.1 | O(16)-Co(19)-O(35)-C(30) | -102.4 |  |  |
| N(27)-Zn(18)-O(34)-C(29) | -19.0 | O(16)-Co(19)-O(35)-C(34) | 92.1 |  |  |
| N(27)-Zn(18)-O(34)-C(33) | -148.4 | N(28)-Co(19)-O(35)-C(30) | -4.7 |  |  |
| O(30)-Zn(18)-O(34)-C(29) | -37.8 | N(28)-Co(19)-O(35)-C(34) | -170.1 |  |  |
| O(30)-Zn(18)-O(34)-C(33) | -167.2 | O(31)-Co(19)-O(35)-C(30) | -28.0 |  |  |
| N(9)-Zn(18)-O(30)-C(25) | 141.3 | O(31)-Co(19)-O(35)-C(34) | 166.5 |  |  |
| O(12)-Zn(18)-O(30)-C(25) | -121.3 | N(9)-Co(19)-O(31)-C(26) | -173.1 |  |  |
| O(16)-Zn(18)-O(30)-C(25) | 79.2 | O(12)-Co(19)-O(31)-C(26) | -88.2 |  |  |
| N(27)-Zn(18)-O(30)-C(25) | -1.2 | O(16)-Co(19)-O(31)-C(26) | 101.3 |  |  |
| O(34)-Zn(18)-O(30)-C(25) | 16.1 | N(28)-Co(19)-O(31)-C(26) | 4.0 |  |  |
| N(9)-Zn(18)-N(27)-N(26) | -126.7 | O(35)-Co(19)-O(31)-C(26) | 27.4 |  |  |
| N(9)-Zn(18)-N(27)-C(28) | 53.3 | N(9)-Co(19)-N(28)-N(27) | 45.4 |  |  |
| O(12)-Zn(18)-N(27)-N(26) | 117.4 | N(9)-Co(19)-N(28)-C(29) | -134.9 |  |  |
| O(12)-Zn(18)-N(27)-C(28) | -62.6 | O(12)-Co(19)-N(28)-N(27) | 91.4 |  |  |
| O(16)-Zn(18)-N(27)-N(26) | -87.9 | O(12)-Co(19)-N(28)-C(29) | -88.9 |  |  |
| O(16)-Zn(18)-N(27)-C(28) | 92.1 | O(16)-Co(19)-N(28)-N(27) | -91.8 |  |  |
| O(30)-Zn(18)-N(27)-N(26) | 1.6 | O(16)-Co(19)-N(28)-C(29) | 87.8 |  |  |
| O(30)-Zn(18)-N(27)-C(28) | -178.4 | O(31)-Co(19)-N(28)-N(27) | -1.5 |  |  |
| O(34)-Zn(18)-N(27)-N(26) | -168.8 | O(31)-Co(19)-N(28)-C(29) | 178.1 |  |  |
| O(34)-Zn(18)-N(27)-C(28) | 11.2 | O(35)-Co(19)-N(28)-N(27) | -177.4 |  |  |
| C(11)-O(16)-Zn(18)-N(9) | -19.0 | O(35)-Co(19)-N(28)-C(29) | 2.3 |  |  |
| C(11)-O(16)-Zn(18)-O(12) | -37.7 | C2(Ph1)-O(17)-H(18)-N(8) | -2.2 |  |  |
| C(11)-O(16)-Zn(18)-N(27) | -171.7 | C(11)-O(16)-Co(19)-N(9) | -3.3 |  |  |
| C(11)-O(16)-Zn(18)-O(30) | 104.7 | C(11)-O(16)-Co(19)-O(12) | -24.0 |  |  |
| C(11)-O(16)-Zn(18)-O(34) | -103.4 | C(11)-O(16)-Co(19)-N(28) | 174.0 |  |  |
| C(15)-O(16)-Zn(18)-N(9) | -148.4 | C(11)-O(16)-Co(19)-O(31) | 89.2 |  |  |
| C(15)-O(16)-Zn(18)-O(12) | -167.1 | C(11)-O(16)-Co(19)-O(35) | -101.0 |  |  |
| C(15)-O(16)-Zn(18)-N(27) | 58.8 | C(15)-O(16)-Co(19)-N(9) | -172.2 |  |  |
| C(15)-O(16)-Zn(18)-O(30) | -24.8 | C(15)-O(16)-Co(19)-O(12) | 167.1 |  |  |
| C(15)-O(16)-Zn(18)-O(34) | 127.1 | C(15)-O(16)-Co(19)-N(28) | 5.1 |  |  |
| C(14)-C(15)-O(16)-C(11) | 0.7 | C(15)-O(16)-Co(19)-O(31) | -79.7 |  |  |
| C(14)-C(15)-O(16)-Zn(18) | 127.7 | C(15)-O(16)-Co(19)-O(35) | 90.1 |  |  |
| H(43)-C(15)-O(16)-C(11) | -179.4 | C(14)-C(15)-O(16)-C(11) | 0.2 |  |  |
| H(43)-C(15)-O(16)-Zn(18) | -52.4 | C(14)-C(15)-O(16)-Co(19) | 168.9 |  |  |
| C(13)-C(14)-C(15)-O(16) | -0.3 | H(44)-C(15)-O(16)-C(11) | -179.6 |  |  |
| C(13)-C(14)-C(15)-H(43) | 179.8 | H(44)-C(15)-O(16)-Co(19) | -10.9 |  |  |
| H(42)-C(14)-C(15)-O(16) | 179.9 | C(13)-C(14)-C(15)-O(16) | -0.1 |  |  |
| H(42)-C(14)-C(15)-H(43) | 0.0 | C(13)-C(14)-C(15)-H(44) | 179.6 |  |  |
| C(11)-C(13)-C(14)-C(15) | -0.2 | H(43)-C(14)-C(15)-O(16) | -179.9 |  |  |
| C(11)-C(13)-C(14)-H(42) | 179.6 | H(43)-C(14)-C(15)-H(44) | -0.1 |  |  |
| H(41)-C(13)-C(14)-C(15) | -179.6 | C(11)-C(13)-C(14)-C(15) | -0.1 |  |  |
| H(41)-C(13)-C(14)-H(42) | 0.2 | C(11)-C(13)-C(14)-H(43) | 179.7 |  |  |
| C(7)-O(12)-Zn(18)-N(9) | -1.2 | H(42)-C(13)-C(14)-C(15) | -179.7 |  |  |
| C(7)-O(12)-Zn(18)-O(16) | 16.0 | H(42)-C(13)-C(14)-H(43) | 0.1 |  |  |
| C(7)-O(12)-Zn(18)-N(27) | 141.3 | C(7)-O(12)-Co(19)-N(9) | 4.1 |  |  |
| C(7)-O(12)-Zn(18)-O(30) | -121.3 | C(7)-O(12)-Co(19)-O(16) | 24.9 |  |  |
| C(7)-O(12)-Zn(18)-O(34) | 79.2 | C(7)-O(12)-Co(19)-N(28) | -173.0 |  |  |
| C(10)-C(11)-O(16)-C(15) | 179.1 | C(7)-O(12)-Co(19)-O(31) | -88.1 |  |  |
| C(10)-C(11)-O(16)-Zn(18) | 24.8 | C(7)-O(12)-Co(19)-O(35) | 101.4 |  |  |
| C(13)-C(11)-O(16)-C(15) | -0.8 | C(10)-C(11)-O(16)-C(15) | 179.1 |  |  |
| C(13)-C(11)-O(16)-Zn(18) | -155.0 | C(10)-C(11)-O(16)-Co(19) | 4.8 |  |  |
| C(10)-C(11)-C(13)-C(14) | -179.2 | C(13)-C(11)-O(16)-C(15) | -0.2 |  |  |
| C(10)-C(11)-C(13)-H(41) | 0.2 | C(13)-C(11)-O(16)-Co(19) | -174.5 |  |  |
| O(16)-C(11)-C(13)-C(14) | 0.6 | C(10)-C(11)-C(13)-C(14) | -179.1 |  |  |
| O(16)-C(11)-C(13)-H(41) | -180.0 | C(10)-C(11)-C(13)-H(42) | 0.6 |  |  |
| N(9)-C(10)-C(11)-C(13) | 155.2 | O(16)-C(11)-C(13)-C(14) | 0.2 |  |  |
| N(9)-C(10)-C(11)-O(16) | -24.7 | O(16)-C(11)-C(13)-H(42) | 179.8 |  |  |
| H(40)-C(10)-C(11)-C(13) | -23.6 | N(9)-C(10)-C(11)-C(13) | 174.9 |  |  |
| H(40)-C(10)-C(11)-O(16) | 156.6 | N(9)-C(10)-C(11)-O(16) | -4.3 |  |  |
| N(8)-N(9)-Zn(18)-O(12) | 1.6 | H(41)-C(10)-C(11)-C(13) | -4.5 |  |  |
| N(8)-N(9)-Zn(18)-O(16) | -168.8 | H(41)-C(10)-C(11)-O(16) | 176.3 |  |  |
| N(8)-N(9)-Zn(18)-N(27) | -126.7 | N(8)-N(9)-Co(19)-O(12) | -1.7 |  |  |
| N(8)-N(9)-Zn(18)-O(30) | 117.4 | N(8)-N(9)-Co(19)-O(16) | -178.0 |  |  |
| N(8)-N(9)-Zn(18)-O(34) | -87.9 | N(8)-N(9)-Co(19)-N(28) | 44.5 |  |  |
| C(10)-N(9)-Zn(18)-O(12) | -178.4 | N(8)-N(9)-Co(19)-O(31) | 91.2 |  |  |
| C(10)-N(9)-Zn(18)-O(16) | 11.1 | N(8)-N(9)-Co(19)-O(35) | -92.5 |  |  |
| C(10)-N(9)-Zn(18)-N(27) | 53.3 | C(10)-N(9)-Co(19)-O(12) | 177.8 |  |  |
| C(10)-N(9)-Zn(18)-O(30) | -62.6 | C(10)-N(9)-Co(19)-O(16) | 1.4 |  |  |
| C(10)-N(9)-Zn(18)-O(34) | 92.0 | C(10)-N(9)-Co(19)-N(28) | -136.1 |  |  |
| N(8)-N(9)-C(10)-C(11) | 179.9 | C(10)-N(9)-Co(19)-O(31) | -89.3 |  |  |
| N(8)-N(9)-C(10)-H(40) | -1.4 | C(10)-N(9)-Co(19)-O(35) | 87.0 |  |  |
| Zn(18)-N(9)-C(10)-C(11) | -0.1 | N(8)-N(9)-C(10)-C(11) | -179.8 |  |  |
| Zn(18)-N(9)-C(10)-H(40) | 178.6 | N(8)-N(9)-C(10)-H(41) | -0.5 |  |  |
| C(7)-N(8)-N(9)-C(10) | 178.3 | Co(19)-N(9)-C(10)-C(11) | 0.9 |  |  |
| C(7)-N(8)-N(9)-Zn(18) | -1.7 | Co(19)-N(9)-C(10)-H(41) | -179.8 |  |  |
| C1(Ph1)-C(7)-O(12)-Zn(18) | -179.0 | C(7)-N(8)-H(18)-O(17) | 2.1 |  |  |
| N(8)-C(7)-O(12)-Zn(18) | 0.7 | N(9)-N(8)-H(18)-O(17) | 173.8 |  |  |
| C1(Ph1)-C(7)-N(8)-N(9) | -179.6 | C(7)-N(8)-N(9)-C(10) | 179.6 |  |  |
| O(12)-C(7)-N(8)-N(9) | 0.7 | C(7)-N(8)-N(9)-Co(19) | -1.0 |  |  |
| C2(Ph1)-C1(Ph1)-C(7)-N(8) | -179.8 | H(18)-N(8)-N(9)-C(10) | 8.4 |  |  |
| C2(Ph1)-C1(Ph1)-C(7)-O(12) | -0.1 | H(18)-N(8)-N(9)-Co(19) | -172.3 |  |  |
| C6(Ph1)-C1(Ph1)-C(7)-N(8) | 0.4 | C1(Ph1)-C(7)-O(12)-Co(19) | 174.7 |  |  |
| C6(Ph1)-C1(Ph1)-C(7)-O(12) | -179.9 | N(8)-C(7)-O(12)-Co(19) | -6.1 |  |  |
| C2(Ph1)-C1(Ph1)-C6(Ph1)-C(1) | 0.3 | C1(Ph1)-C(7)-N(8)-N(9) | -175.7 |  |  |
| C2(Ph1)-C1(Ph1)-C6(Ph1)-H(39) | -179.6 | C1(Ph1)-C(7)-N(8)-H(18) | -0.6 |  |  |
| C(7)-C1(Ph1)-C6(Ph1)-C(1) | -179.9 | O(12)-C(7)-N(8)-N(9) | 5.1 |  |  |
| C(7)-C1(Ph1)-C6(Ph1)-H(39) | 0.2 | O(12)-C(7)-N(8)-H(18) | -179.8 |  |  |
| C3(Ph1)-C2(Ph1)-O(17)-H(44) | 179.0 | C2(Ph1)-C1(Ph1)-C(7)-N(8) | 0.4 |  |  |
| C1(Ph1)-C2(Ph1)-O(17)-H(44) | -1.1 | C2(Ph1)-C1(Ph1)-C(7)-O(12) | 179.6 |  |  |
| C3(Ph1)-C2(Ph1)-C1(Ph1)-C6(Ph1) | -0.4 | C6(Ph1)-C1(Ph1)-C(7)-N(8) | 180.0 |  |  |
| C3(Ph1)-C2(Ph1)-C1(Ph1)-C(7) | 179.8 | C6(Ph1)-C1(Ph1)-C(7)-O(12) | -0.8 |  |  |
| O(17)-C2(Ph1)-C1(Ph1)-C6(Ph1) | 179.8 | C2(Ph1)-C1(Ph1)-C6(Ph1)-C(1) | -0.1 |  |  |
| O(17)-C2(Ph1)-C1(Ph1)-C(7) | 0.0 | C2(Ph1)-C1(Ph1)-C6(Ph1)-H(40) | 179.8 |  |  |
| C(2)-C3(Ph1)-C2(Ph1)-C1(Ph1) | 0.2 | C(7)-C1(Ph1)-C6(Ph1)-C(1) | -179.6 |  |  |
| C(2)-C3(Ph1)-C2(Ph1)-O(17) | -179.9 | C(7)-C1(Ph1)-C6(Ph1)-H(40) | 0.2 |  |  |
| H(38)-C3(Ph1)-C2(Ph1)-C1(Ph1) | -179.9 | C3(Ph1)-C2(Ph1)-O(17)-H(18) | -179.0 |  |  |
| H(38)-C3(Ph1)-C2(Ph1)-O(17) | -0.1 | C1(Ph1)-C2(Ph1)-O(17)-H(18) | 1.1 |  |  |
| C(1)-C(2)-C3(Ph1)-C2(Ph1) | 0.0 | C3(Ph1)-C2(Ph1)-C1(Ph1)-C6(Ph1) | 0.1 |  |  |
| C(1)-C(2)-C3(Ph1)-H(38) | -179.8 | C3(Ph1)-C2(Ph1)-C1(Ph1)-C(7) | 179.6 |  |  |
| H(37)-C(2)-C3(Ph1)-C2(Ph1) | 179.9 | O(17)-C2(Ph1)-C1(Ph1)-C6(Ph1) | 180.0 |  |  |
| H(37)-C(2)-C3(Ph1)-H(38) | 0.1 | O(17)-C2(Ph1)-C1(Ph1)-C(7) | -0.5 |  |  |
| C(2)-C(1)-C6(Ph1)-C1(Ph1) | -0.1 | C(2)-C3(Ph1)-C2(Ph1)-C1(Ph1) | 0.0 |  |  |
| C(2)-C(1)-C6(Ph1)-H(39) | 179.8 | C(2)-C3(Ph1)-C2(Ph1)-O(17) | -179.9 |  |  |
| H(36)-C(1)-C6(Ph1)-C1(Ph1) | -180.0 | H(39)-C3(Ph1)-C2(Ph1)-C1(Ph1) | -179.9 |  |  |
| H(36)-C(1)-C6(Ph1)-H(39) | 0.0 | H(39)-C3(Ph1)-C2(Ph1)-O(17) | 0.2 |  |  |
| C6(Ph1)-C(1)-C(2)-C3(Ph1) | 0.0 | C(1)-C(2)-C3(Ph1)-C2(Ph1) | 0.0 |  |  |
| C6(Ph1)-C(1)-C(2)-H(37) | -180.0 | C(1)-C(2)-C3(Ph1)-H(39) | 179.9 |  |  |
| H(36)-C(1)-C(2)-C3(Ph1) | 179.8 | H(38)-C(2)-C3(Ph1)-C2(Ph1) | -179.9 |  |  |
| H(36)-C(1)-C(2)-H(37) | -0.1 | H(38)-C(2)-C3(Ph1)-H(39) | 0.0 |  |  |
|  |  | C(2)-C(1)-C6(Ph1)-C1(Ph1) | 0.0 |  |  |
|  |  | C(2)-C(1)-C6(Ph1)-H(40) | -179.8 |  |  |
|  |  | H(37)-C(1)-C6(Ph1)-C1(Ph1) | 179.9 |  |  |
|  |  | H(37)-C(1)-C6(Ph1)-H(40) | 0.1 |  |  |
|  |  | C6(Ph1)-C(1)-C(2)-C3(Ph1) | 0.0 |  |  |
|  |  | C6(Ph1)-C(1)-C(2)-H(38) | 179.9 |  |  |
|  |  | H(37)-C(1)-C(2)-C3(Ph1) | -179.9 |  |  |
|  |  | H(37)-C(1)-C(2)-H(38) | 0.1 |  |  |

***Cartesian coordinates ofCu(II) complex***

Center Atomic Atomic Coordinates (Angstroms)

Number Number Type X Y Z

---------------------------------------------------------------------

1 6 0 -5.559905 2.291994 -1.735227

2 6 0 -6.203224 1.177862 -2.275855

3 6 0 -5.562894 -0.049271 -2.312805

4 6 0 -4.256148 -0.168801 -1.802031

5 6 0 -3.592465 0.954207 -1.255726

6 6 0 -4.272472 2.182314 -1.232755

7 6 0 -2.221863 0.862743 -0.725923

8 7 0 -1.604727 1.966156 -0.251740

9 7 0 -0.373862 1.822234 0.339910

10 8 0 -1.623295 -0.307479 -0.743520

11 8 0 -3.724693 -1.415974 -1.882037

12 6 0 0.167439 3.008486 0.745100

13 6 0 1.374967 3.202645 1.466572

14 6 0 1.965794 4.432037 1.813834

15 6 0 3.143987 4.121418 2.551023

16 6 0 3.212209 2.738380 2.620993

17 8 0 2.148880 2.161578 1.971486

18 1 0 -2.837018 -1.396683 -1.523076

19 6 0 5.457935 -2.767859 -1.477936

20 6 0 6.153294 -1.756585 -2.141838

21 6 0 5.610753 -0.487869 -2.246312

22 6 0 4.350622 -0.220353 -1.678180

23 6 0 3.634460 -1.238246 -1.007508

24 6 0 4.215274 -2.513296 -0.920176

25 6 0 2.306267 -0.992618 -0.420057

26 7 0 1.616942 -2.013525 0.128717

27 7 0 0.350675 -1.754623 0.629328

28 8 0 1.850764 0.243395 -0.413162

29 8 0 3.913512 1.055251 -1.835194

30 6 0 -0.190002 -2.763079 1.314674

31 6 0 -1.466988 -2.793957 1.969045

32 6 0 -2.113456 -3.906740 2.508566

33 6 0 -3.337295 -3.431375 3.073199

34 6 0 -3.365517 -2.067650 2.852629

35 8 0 -2.238464 -1.657964 2.185682

36 1 0 3.055120 1.141677 -1.418725

37 29 0 0.048257 0.013412 0.035636

38 1 0 -6.074066 3.258088 -1.709795

39 1 0 -7.219149 1.274399 -2.673220

40 1 0 -6.064660 -0.926704 -2.737416

41 1 0 -3.782338 3.070756 -0.814847

42 1 0 -0.388187 3.940909 0.535813

43 1 0 1.595595 5.423248 1.565209

44 1 0 3.847134 4.835071 2.971829

45 1 0 3.913151 2.039603 3.067793

46 1 0 5.895659 -3.768188 -1.399638

47 1 0 7.133332 -1.967988 -2.582690

48 1 0 6.153566 0.309060 -2.768079

49 1 0 3.682145 -3.322694 -0.405255

50 1 0 0.373423 -3.710426 1.406639

51 1 0 -1.761694 -4.935206 2.501069

52 1 0 -4.089782 -4.034335 3.573774

53 1 0 -4.073146 -1.278011 3.087781

---------------------------------------------------------------------

***Cartesian coordinates ofNi(II) complex***

---------------------------------------------------------------------

Center Atomic Atomic Coordinates (Angstroms)

Number Number Type X Y Z

---------------------------------------------------------------------

1 6 0 4.987432 -2.055647 -2.615664

2 6 0 5.834101 -2.474866 -1.588369

3 6 0 5.541328 -2.169652 -0.270820

4 6 0 4.381251 -1.430927 0.030516

5 6 0 3.515979 -1.002928 -1.001594

6 6 0 3.843345 -1.330048 -2.327221

7 6 0 2.297435 -0.243281 -0.716144

8 7 0 1.522823 0.256140 -1.699437

9 7 0 0.357862 0.892500 -1.284088

10 8 0 1.994370 0.031220 0.540041

11 8 0 4.186814 -1.180287 1.349997

12 6 0 -0.202669 1.698807 -2.170821

13 6 0 -1.431331 2.429241 -1.978259

14 6 0 -2.550910 2.254434 -1.178234

15 6 0 -3.455297 3.315785 -1.511609

16 6 0 -2.839614 4.065328 -2.491396

17 8 0 -1.607481 3.544839 -2.799917

18 1 0 3.385326 -0.666576 1.454516

19 6 0 -5.187606 -2.906502 1.326634

20 6 0 -5.453815 -3.343663 0.029020

21 6 0 -4.566362 -3.072364 -0.998233

22 6 0 -3.393571 -2.341361 -0.730583

23 6 0 -3.127619 -1.881577 0.572708

24 6 0 -4.033882 -2.187992 1.596712

25 6 0 -1.932868 -1.091922 0.901749

26 7 0 -1.162367 -1.409258 1.969472

27 7 0 -0.113704 -0.529729 2.068842

28 8 0 -1.686177 0.008879 0.208978

29 8 0 -2.588657 -2.150401 -1.811574

30 6 0 0.312522 0.171221 3.098803

31 6 0 0.908535 1.432360 2.724136

32 6 0 1.640432 2.350526 3.457308

33 6 0 1.734878 3.535537 2.660140

34 6 0 1.065703 3.286423 1.483583

35 8 0 0.577060 1.971451 1.459576

36 1 0 -1.786889 -1.722910 -1.543488

37 28 0 0.142045 0.252085 0.443686

38 1 0 5.229028 -2.302307 -3.654564

39 1 0 6.735910 -3.049016 -1.826625

40 1 0 6.204409 -2.497706 0.538040

41 1 0 3.186878 -1.008128 -3.145789

42 1 0 0.292470 1.844255 -3.148479

43 1 0 -2.729675 1.448475 -0.453856

44 1 0 -4.434524 3.479906 -1.070883

45 1 0 -3.119255 4.952641 -3.051262

46 1 0 -5.890848 -3.133900 2.134175

47 1 0 -6.368680 -3.909356 -0.177972

48 1 0 -4.769693 -3.424211 -2.016593

49 1 0 -3.826769 -1.854963 2.620672

50 1 0 0.155668 -0.076326 4.157058

51 1 0 2.059055 2.210708 4.450799

52 1 0 2.239824 4.454396 2.947365

53 1 0 0.896390 3.881862 0.589168

---------------------------------------------------------------------

***\***

***Cartesian coordinates ofZn(II) complex***

---------------------------------------------------------------------

Center Atomic Atomic Coordinates (Angstroms)

Number Number Type X Y Z

---------------------------------------------------------------------

1 6 0 -5.747153 -0.531329 -2.107178

2 6 0 -5.769444 -1.703138 -2.864695

3 6 0 -4.609959 -2.433612 -3.055069

4 6 0 -3.404161 -1.988785 -2.478900

5 6 0 -3.368612 -0.803705 -1.707590

6 6 0 -4.564483 -0.087044 -1.537735

7 6 0 -2.120641 -0.313132 -1.088362

8 7 0 -2.152468 0.847524 -0.370517

9 7 0 -0.975326 1.264174 0.191332

10 8 0 -1.023788 -1.010404 -1.248555

11 8 0 -2.327096 -2.775652 -2.724017

12 6 0 -0.859545 2.386573 0.885684

13 6 0 -1.846956 3.377037 1.207548

14 6 0 -1.665497 4.557565 1.934649

15 6 0 -2.938234 5.201649 1.984284

16 6 0 -3.815671 4.386110 1.291965

17 8 0 -3.174790 3.273887 0.812376

18 1 0 -1.561636 -2.382580 -2.297621

19 6 0 6.997361 -0.230426 -0.166901

20 6 0 7.343219 0.506478 -1.300426

21 6 0 6.359055 1.050589 -2.106094

22 6 0 5.003098 0.857646 -1.776548

23 6 0 4.639138 0.113059 -0.630541

24 6 0 5.665902 -0.424936 0.162761

25 6 0 3.226064 -0.109124 -0.258962

26 7 0 2.945026 -0.860373 0.833851

27 7 0 1.614575 -1.078894 1.198387

28 8 0 2.290046 0.438618 -1.000678

29 8 0 4.119751 1.433489 -2.629435

30 6 0 1.499489 -1.858281 2.267529

31 6 0 0.316365 -2.291608 2.958033

32 6 0 0.261011 -3.178480 4.037247

33 6 0 -1.116773 -3.302642 4.392547

34 6 0 -1.819682 -2.491310 3.521541

35 8 0 -0.969722 -1.867171 2.642915

36 1 0 3.230964 1.235716 -2.324837

37 30 0 0.495052 -0.074278 -0.222198

38 1 0 -6.669226 0.040780 -1.963605

39 1 0 -6.708994 -2.045520 -3.311182

40 1 0 -4.622099 -3.353651 -3.650889

41 1 0 -4.568864 0.838871 -0.947788

42 1 0 0.151575 2.595170 1.272883

43 1 0 -0.740248 4.919377 2.375126

44 1 0 -3.161576 6.146586 2.471573

45 1 0 -4.874315 4.446264 1.057315

46 1 0 7.783008 -0.657721 0.464425

47 1 0 8.398502 0.654235 -1.553344

48 1 0 6.625537 1.629203 -2.998170

49 1 0 5.417923 -1.009459 1.057912

50 1 0 2.420289 -2.260939 2.736542

51 1 0 1.099375 -3.679929 4.514857

52 1 0 -1.521070 -3.918141 5.191744

53 1 0 -2.871910 -2.253989 3.390077

---------------------------------------------------------------------

***Cartesian coordinates ofCo(II) complex***

---------------------------------------------------------------------

Center Atomic Atomic Coordinates (Angstroms)

Number Number Type X Y Z

---------------------------------------------------------------------

1 6 0 4.581613 3.223392 1.466583

2 6 0 5.527372 3.485857 0.445237

3 6 0 5.422053 2.864639 -0.805424

4 6 0 4.365246 1.963965 -1.063400

5 6 0 3.406482 1.688920 -0.038301

6 6 0 3.533960 2.331836 1.218130

7 6 0 2.292451 0.754903 -0.258414

8 7 0 2.203928 0.169156 -1.495465

9 7 0 1.113180 -0.680090 -1.549816

10 6 0 0.847098 -1.371212 -2.632779

11 6 0 -0.284862 -2.259299 -2.599061

12 8 0 1.429602 0.487180 0.690064

13 6 0 -0.948679 -3.065945 -3.503500

14 6 0 -2.045148 -3.691424 -2.798435

15 6 0 -2.006969 -3.248068 -1.498126

16 8 0 -0.924195 -2.370706 -1.339227

17 8 0 4.302758 1.380952 -2.306017

18 1 0 3.487507 0.773629 -2.338275

19 27 0 0.000093 -0.729195 -0.002090

20 6 0 -4.582442 3.226700 -1.459732

21 6 0 -5.526136 3.488592 -0.436346

22 6 0 -5.419580 2.865033 0.813043

23 6 0 -4.363634 1.962408 1.067819

24 6 0 -3.406979 1.687882 0.040511

25 6 0 -3.535550 2.333334 -1.214482

26 6 0 -2.293653 0.752204 0.257123

27 7 0 -2.204310 0.163915 1.492845

28 7 0 -1.113151 -0.685055 1.545731

29 6 0 -0.848452 -1.378707 2.627377

30 6 0 0.283834 -2.266382 2.594546

31 8 0 -1.431146 0.486950 -0.692332

32 6 0 0.943122 -3.076851 3.498970

33 6 0 2.042708 -3.699826 2.796635

34 6 0 2.009642 -3.252513 1.497494

35 8 0 0.928566 -2.373443 1.337260

36 8 0 -4.299772 1.377154 2.309216

37 1 0 4.668521 3.712943 2.432420

38 1 0 6.345295 4.178937 0.629553

39 1 0 6.136274 3.056776 -1.600033

40 1 0 2.789557 2.110466 1.977270

41 1 0 1.454294 -1.282969 -3.528851

42 1 0 -0.699025 -3.187723 -4.547315

43 1 0 -2.769235 -4.377310 -3.212776

44 1 0 -2.609027 -3.424082 -0.621188

45 1 0 -4.670284 3.718105 -2.424540

46 1 0 -6.343447 4.183095 -0.618040

47 1 0 -6.132272 3.056856 1.609112

48 1 0 -2.792619 2.112350 -1.975171

49 1 0 -1.457348 -1.292743 3.522538

50 1 0 0.688703 -3.202375 4.541195

51 1 0 2.764933 -4.387234 3.211699

52 1 0 2.615392 -3.425712 0.622535

53 1 0 -3.484736 0.768436 2.338691

---------------------------------------------------------------------

***Cartesian coordinates ofligand***

---------------------------------------------------------------------

Center Atomic Atomic Coordinates (Angstroms)

Number Number Type X Y Z

---------------------------------------------------------------------

1 6 0 -4.415479 1.150685 -0.141259

2 6 0 -3.489953 0.140184 -0.016800

3 6 0 -5.715695 0.548551 -0.100549

4 6 0 -5.524864 -0.795197 0.045598

5 8 0 -4.166667 -1.078351 0.100045

6 6 0 -2.055013 0.185106 0.005359

7 7 0 -1.360764 -0.908163 0.135913

8 7 0 0.030657 -0.885317 0.148823

9 6 0 0.911689 0.152688 0.001282

10 8 0 0.492778 1.354696 -0.085792

11 6 0 4.230511 -1.723446 -0.133380

12 6 0 5.131804 -0.651018 -0.024525

13 6 0 2.865943 -1.471542 -0.138457

14 6 0 4.664346 0.652186 0.060356

15 6 0 2.356558 -0.157933 -0.032454

16 6 0 3.286510 0.913206 0.046842

17 8 0 2.904873 2.228253 0.110537

18 1 0 -4.192752 2.198222 -0.248250

19 1 0 -6.662159 1.054667 -0.171379

20 1 0 -6.180936 -1.640435 0.123854

21 1 0 -1.599225 1.160583 -0.090584

22 1 0 0.384387 -1.814785 0.285566

23 1 0 4.594545 -2.737500 -0.220192

24 1 0 6.197154 -0.839506 -0.017814

25 1 0 2.193512 -2.312452 -0.250081

26 1 0 5.335404 1.495832 0.130143

27 1 0 1.914806 2.292129 0.035196

---------------------------------------------------------------------
